# From product scoring to online language processing: associations of AI-supported process feedback with pausing and revision trajectories in L2 writing

**DOI:** 10.3389/fpsyg.2026.1951427

**Published:** 2026-10-21

**Authors:** Jiaojiao Wu, Zalina Mohd Kasim, Fatin Nabila Abd Razak

**Affiliations:** 1 Faculty of International Education, Sias University, Zhengzhou, China; 2 Faculty of Modern Languages and Communication, Universiti Putra Malaysia, Serdang, Malaysia

**Keywords:** AI-supported feedback, automated writing evaluation, keystroke logging, metalinguistic monitoring, pausing behavior, revision, second language writing, transfer

## Abstract

**Introduction:**

AI-supported writing feedback can influence how second language (L2) learners compose, monitor, and revise their texts. This study examined how feedback delivered during writing relates to learners' writing processes and their subsequent performance without AI assistance.

**Methods:**

A longitudinal experiment randomly assigned 297 Chinese university learners of English as a foreign language to three conditions: rubric-based control, AI product feedback, and AI process feedback. Participants completed a baseline writing task, two intervention tasks, and a delayed no-AI transfer task. Product feedback was provided after drafting, whereas process feedback was delivered during composing. Keystroke records, pausing patterns, production bursts, revision behavior, metalinguistic monitoring, and independently rated writing quality were analyzed to examine changes in writing processes and performance.

**Results:**

Compared with product feedback, process feedback was associated with a lower proportion of within-clause pauses and a higher proportion of boundary pauses during the second intervention. Differences in boundary pausing and higher-level revision remained evident during the no-AI transfer task. The process-feedback group also achieved higher adjusted transfer scores than the product-feedback group (+1.951 points, 95% CI [0.414, 3.489], *p* = 0.013), with a standardized effect of *d* = 0.376 (bootstrap 95% CI [0.096, 0.680]). Process feedback was associated with higher cognitive-load ratings during the first intervention compared with control. Metalinguistic monitoring also showed a statistically reliable indirect association with transfer performance.

**Discussion:**

AI process feedback was associated with changes in how learners distributed attention during writing, including greater pausing at structural boundaries and more higher-level revision. These patterns were accompanied by stronger performance in subsequent independent writing. The findings suggest that delivering feedback during composing can support learners' ongoing monitoring and revision decisions, with benefits extending to writing tasks completed without AI assistance.

## 1 Introduction

Second language writing research has long relied on final products because texts are stable, scoreable, and closely tied to classroom decisions. Analytic ratings, error-free clause proportions, lexical measures, syntactic indices, and cohesion scores capture important properties of the submitted text while leaving the route through which the learner composed it largely unobserved. Two essays with similar scores can emerge from different processes: one writer may produce long stretches of language before rereading and revising globally, while another may interrupt nearly every clause to search for vocabulary, repair grammar, and rebuild syntax. Cognitive models of writing describe composing as an interaction among planning, translating, reviewing, and monitoring (Flower and Hayes, 1981; Hayes, 2012). Working-memory accounts further suggest that L2 writers' limited attentional resources make the coordination of planning, linguistic encoding, transcription, and revision fragile (Kellogg, 1996; McCutchen, 1996). Final scores record the surviving text while leaving the allocation of attention during composing largely outside the rating.

Keystroke logging records writing as it unfolds. Timestamps, cursor movements, pause locations, production bursts, and revision sequences show how much learners write, when they stop, where they stop, and what follows each interruption (Leijten and Van Waes, 2013; Baaijen et al., 2012). External interruptions make the timing of resumption important because writers return to suspended tasks with different goals and supporting cues (Altmann and Trafton, 2002; Trafton et al., 2003). Recent online-writing research likewise treats feedback and regulation as temporally situated activities alongside the final text (Bowen et al., 2022). Pause location separates activity inside words and clauses from activity at sentence or paragraph boundaries, while production-burst length records continuity of language execution. Proficiency, task demands, typing fluency, and revision strategy provide the context for interpreting these streams (Révész et al., 2017; Medimorec and Risko, 2017; Van Waes and Leijten, 2015; Conijn et al., 2019). Process data therefore become most informative when pause location, burst length, revision, metalinguistic judgement, and later text quality are examined together.

Revision evidence connects text outcomes with the decisions made during composing. Deletions and replacements capture local editing activity, while delayed revisions that alter clause relations, sentence meaning, paragraph structure, or argument coherence capture higher-level restructuring. Studies of L2 revision emphasize problem detection, diagnosis, alternative generation, and evaluation of the revised text (Barkaoui, 2007; Ferris, 2010; Storch, 2018). Feedback research reaches a related conclusion: learners notice a comment, identify its target, judge its relevance, and integrate it into the text (Hyland and Hyland, 2006; Zhang and Hyland, 2018). AI-supported feedback consequently needs evidence about how comments engage learners' own monitoring while they write.

Automated writing evaluation (AWE) and generative AI systems have widened access to feedback on grammar, organization, vocabulary, and style. Earlier AWE work framed automated systems as classroom supports whose value depends on feedback quality and the instructional context in which learners engage with comments (Warschauer and Ware, 2006; Warschauer and Grimes, 2008; Li et al., 2015). Studies of automated written corrective feedback show that uptake varies across learners and error types (Ranalli, 2018; Koltovskaia, 2020; Zhang, 2020). ChatGPT and related systems can now provide fluent explanations, reformulations, and metalinguistic comments, and this flexibility also makes it harder to distinguish learning-oriented feedback from replacement language (Barrot, 2023; Kang and Yi, 2023; Hyland, 2026). Recent empirical studies and meta-analytic work report positive effects of AI-supported writing feedback, while stressing feedback literacy, teacher mediation, actionability, and critical AI literacy (Li, 2024; Yeung, 2025; Kashiha, 2025; Wang and Wang, 2025; Darvin, 2025; Luo and Zhan, 2026). Evidence from adjacent educational settings is more mixed: a randomized study reported no short-term learning advantage from ChatGPT use, whereas a field experiment found poorer unaided performance after unrestricted access to GPT-4 practice support (Svendsen et al., 2024; Bastani et al., 2025).

The central gap concerns feedback orientation and the moment at which feedback enters the writing process. Product feedback arrives after a draft or text segment has been formed and supports diagnosis of the visible product. Process feedback uses behavioral signals during writing to prompt inspection within the ongoing episode. These packages differ in timing, exposure, message length, and opportunities for immediate action, making their behavioral profiles an empirical question. Studies on AI-adaptive feedback and teacher-moderated ChatGPT feedback in L2 writing already point toward the importance of feedback engagement and revision mediation (Luo and Yusuf, 2026; Pretorius and Thewissen, 2026; Zhou et al., 2026); the unresolved issue is whether process-oriented support is associated with a different organization of pausing and revising than product-oriented support.

To examine that gap, 297 Chinese university EFL learners were assigned to rubric-based control, AI product feedback, or AI process feedback across a baseline task, two intervention tasks, and a delayed no-AI transfer task. The analysis combines task-level outcomes with 1,095,390 keystroke action events, 106,470 pause events, 23,374 revision events, 773 feedback events, metalinguistic monitoring responses, and blinded text ratings. It asks whether process feedback is associated with changes in pause location and production bursts, revision level and effectiveness, and transfer writing quality, and whether the transfer contrast is statistically related to metalinguistic monitoring and higher-level revision. The no-AI transfer task extends the comparison to independent writing after the feedback-supported sessions.

Three research questions guided the analysis. RQ1 asked whether AI process feedback is associated with differences in pausing and production-burst trajectories across writing sessions. RQ2 asked whether process feedback is associated with differences in revision level and effectiveness across sessions, with revision quantity, delayed status, revision distance, and feedback-window timing providing complementary process evidence. RQ3 asked whether transfer writing quality is statistically associated with metalinguistic monitoring and higher-level revision after controlling for baseline quality, English proficiency, typing speed, and prompt assignment (Supplementary Table S5).

## 2 Related work and theoretical framework

### 2.1 Product-oriented assessment and process evidence in L2 writing

Product-oriented assessment remains indispensable in L2 writing because educational decisions require reliable judgments of final performance. Analytic rating, corrective feedback, and text-quality indices reveal whether a final text is accurate, complex, coherent, and appropriate for the task. Written corrective feedback research has established that targeted comments can improve selected linguistic outcomes when learners have opportunities to revise and when feedback is tied to instruction (Ferris, 1999; Bitchener and Knoch, 2010; Ferris, 2010). Teacher-feedback and sociocultural perspectives add a process requirement: learners interpret comments, negotiate their relevance, regulate attention, and revise under specific instructional conditions (Hyland and Hyland, 2006; Storch, 2018). AI feedback therefore raises a familiar problem in a new form, since text improvement and learner monitoring can diverge.

Final texts omit abandoned plans, deleted clauses, repeated attempts, feedback reading time, and the location of monitoring. Product scores can therefore conceal distinct routes to the same outcome. Keystroke logging and process-tracing methods complement product assessment by exposing the temporal path through which the product was made (Leijten and Van Waes, 2013; Vandermeulen et al., 2020). Process evidence links text quality with the organization of production and helps distinguish short-term editing assistance from patterns that continue into writing without immediate feedback.

L2 writing complexity and AI-supported composing both require this dynamic view. Linguistic complexity changes with task, proficiency, and repeated writing occasions (Révész et al., 2017; Wang and Tao, 2026). ChatGPT use in the pre-writing stage also links learners' affective engagement and writing performance to how they use AI support during composing (Nguyen et al., 2025). Once feedback enters an ongoing writing process, higher final quality must be examined alongside the writer's pause locations, production bursts, revision decisions, and metalinguistic monitoring. The present analysis treats those measures as linked evidence streams.

### 2.2 Pausing, production bursts, and revision as evidence of monitoring

Pause location and surrounding behavior provide the context for interpreting process evidence. A pause inside a clause may follow a lexical search or syntactic construction problem; a pause at a sentence boundary may precede a shift in proposition, a check of coherence, or planning for the next move. Medimorec and Risko showed that where writers pause can be as important as how long they pause (Medimorec and Risko, 2017). Keystroke studies similarly argue that process measures should be located in the writing episode and interpreted alongside output features (Baaijen et al., 2012; Conijn et al., 2019). Bowen et al. further used keystroke logging to examine the temporal relation between feedback and regulation in online writing classes (Bowen et al., 2022). For L2 writers, pausing can mark difficulty or strategic regulation, depending on whether the writer is struggling with language encoding or checking a claim, transition, or sentence relation.

The interval between pauses adds a second view of online production because burst length records how long writers continue before the next interruption. Longer bursts can reflect fluent encoding, formulaic production, or postponed revision, while shorter bursts can reflect low fluency, close monitoring, or a difficult rhetorical move. Van Waes and Leijten proposed a multidimensional view of writing fluency that separates temporal continuity from text quantity and revision behavior (Van Waes and Leijten, 2015). In this analysis, mean burst length is interpreted with within-clause pause proportion, boundary pause proportion, and higher-level revision, so changes in continuous production are not reduced to words per minute.

Revision research makes the same surface-function distinction. Barkaoui's review of L2 revision highlights the need to distinguish types, levels, and outcomes of revision instead of treating all changes as equivalent (Barkaoui, 2007). Automated and teacher-feedback studies show that engagement with feedback appears in revision behavior, with uptake quality differing across learners and feedback types (Zhang and Hyland, 2018; Koltovskaia, 2020; Zhang, 2020). The present coding separates higher-level revision, defined as revision at clause level or above, from local revision; it also separates effective revisions from changes that fail to improve the text or introduce new problems.

### 2.3 AI feedback, feedback literacy, and the process question

AI-supported feedback inherits the strengths and vulnerabilities of earlier AWE. Automated systems can provide rapid comments, but precision varies across error categories, and students may need to judge whether a suggestion is accurate before using it (Warschauer and Ware, 2006; Li et al., 2015; Ranalli, 2018; Bai and Hu, 2017). Generative AI increases flexibility because it can explain, diagnose, and tailor comments across rhetorical and linguistic dimensions. A comparison of ChatGPT-generated and peer feedback on argumentative essays also found different feedback profiles and no overall association between essay quality and feedback quality, which supports treating feedback quality as an empirical property that requires independent evaluation (Banihashem et al., 2024). It can also supply fluent text that learners may accept without enough evaluation. Barrot described the potential and pitfalls of ChatGPT for L2 writing (Barrot, 2023), and Kang and Yi argued that multimodal generative AI expands the ecology of L2 composing beyond text-only feedback (Kang and Yi, 2023). Process-based evaluation asks how AI support relates both to the current draft and to writers' subsequent monitoring without AI support.

Recent L2 writing studies locate that question in feedback engagement and feedback literacy. Li discussed ChatGPT as a tool for L2 writing feedback and assessment (Li, 2024); Yeung examined university students' engagement with generative AI-supported AWE feedback (Yeung, 2025); Kashiha compared AI and human feedback practices in academic writing (Kashiha, 2025). Studies that combine ChatGPT with teacher feedback report an instructional arrangement in which automated explanations are interpreted and extended through teacher guidance (Asadi et al., 2025a); related work on AI-assisted teacher instruction examines AI as part of an organized writing intervention rather than as a stand-alone comment generator (Asadi et al., 2025b). These approaches differ from AWE, which primarily scores or diagnoses the completed text, and from peer/AI-supported assessment, which emphasizes evaluative participation and engagement. Critical AI literacy studies add that learners need the ability to interrogate AI output and resist passive acceptance (Wang and Wang, 2025; Darvin, 2025). Teacher-related work further shows that feedback literacy depends on teachers' knowledge, values, and abilities as well as learners' uptake (Lee and Mao, 2024). Together, these strands position process feedback as a distinct architecture: automated diagnostic information is delivered during composing, while the learner remains responsible for evaluating and applying it.

Broader AI-in-education reviews frame the same methodological issue at field level. Zawacki-Richter et al. found that AI applications in higher education often foreground technical affordances while underrepresenting educators and learning processes (Zawacki-Richter et al., 2019). Kasneci et al. and Tlili et al. outlined opportunities and risks of large language models in education (Kasneci et al., 2023; Tlili et al., 2023); Bond et al. called for greater rigor, ethics, and collaboration in AI higher education research (Bond et al., 2024). A broader risk taxonomy includes misinformation and human–computer interaction harms, both of which make uncritical acceptance and dependence relevant concerns for feedback systems (Weidinger et al., 2022). In L2 writing, AI feedback research now needs to specify the learning process being supported. Process feedback is theorized here as external metalinguistic monitoring: a prompt that draws attention to the learner's current composing state and invites diagnosis without supplying replacement text.

### 2.4 Hypothesized mechanism and directional hypotheses

The theoretical model guiding the study treats L2 writing as a recursive system in which writers coordinate planning, linguistic encoding, transcription, monitoring, and revision under limited attention. AI process feedback was expected to reorganize observable composing activity by directing attention to a current process problem without supplying replacement wording. The model generated directional predictions for pause location, revision, and independent transfer. Keystroke variables were defined as textual and temporal indicators, with cognitive interpretation grounded in the combined pattern of process, monitoring, and performance measures.

**H1**. Relative to the control and product-feedback conditions, the process-feedback condition was expected to show a lower proportion of within-clause pauses and a higher proportion of sentence- and paragraph-boundary pauses across the intervention sequence. A longer mean production-burst length was a secondary directional prediction, because burst length can also reflect fluency and the timing of postponed revision.
**H2**. Relative to the control and product-feedback conditions, the process-feedback condition was expected to show higher proportions of higher-level revision and effective revision during the intervention and transfer tasks. Revision count, delayed status, revision distance, and feedback-window timing provided complementary process evidence.
**H3**. The process-feedback condition was expected to obtain higher writing quality on the delayed no-AI transfer task than the control and product-feedback conditions. Metalinguistic monitoring was expected to account statistically for part of this contrast. The monitoring and revision pathways were examined as statistical associations within the longitudinal design.

The full sequence from process feedback to Intervention 1 monitoring, Intervention 2 higher-level revision, and no-AI transfer quality was examined as a secondary pathway. The analysis distinguished the monitoring pathway, the revision pathway, and the complete sequential pathway, with bootstrap intervals used to characterize each association.

## 3 Methods

### 3.1 Participants and experimental design

Participants were recruited from university English courses at Zhengzhou Sias University between March 2 and March 13, 2026. The sample comprised 297 full-time, non-English-major undergraduates whose primary language was Chinese. Their mean age was 19.98 years (SD = 1.13, range = 18–23); 189 participants (63.6%) were women and 108 (36.4%) were men. They had studied English for a mean of 10.4 years (SD = 1.8), and their prior English writing experience included preparation for the National College Entrance Examination (Gaokao). English proficiency was measured with the institution's standardized University English Proficiency Test (0–100); the overall mean was 67.62 (SD = 9.72), with condition means reported in Table 1. Eligible students were aged 18 years or older and could complete English argumentative writing independently. Students were excluded if they did not complete the baseline writing task, had duplicated registration, or could not provide written consent. No financial compensation was offered, and participation did not affect course grades.

**Table 1:** Participant retention and baseline characteristics in the randomized sample (*N* = 297).

Measure	Control	Product feedback	Process feedback	Max. |SMD|
Randomized, n	99	99	99	–
Completed baseline, n	99	99	99	–
Completed Intervention 1, n	97	97	98	–
Completed Intervention 2, n	95	94	96	–
Completed transfer, n	93	92	94	–
Completed all sessions, n	93	92	94	–
Age, years	19.94 ± 1.22	20.02 ± 1.08	19.99 ± 1.10	0.070
Female, n (%)	68 (68.7)	56 (56.6)	65 (65.7)	0.253
English proficiency, 0–100	66.56 ± 10.10	68.60 ± 10.25	67.69 ± 8.74	0.200
Typing speed, WPM	36.46 ± 8.68	37.43 ± 8.83	37.09 ± 8.43	0.112
Prior AI use, 0–5	2.84 ± 1.15	2.69 ± 1.15	2.70 ± 1.12	0.137
Baseline writing quality	63.46 ± 9.33	64.63 ± 9.30	64.35 ± 8.95	0.126
Baseline metalinguistic score, 0–24	12.06 ± 4.83	12.17 ± 5.22	11.74 ± 4.45	0.090
Baseline within-clause pause proportion	0.28 ± 0.05	0.28 ± 0.05	0.29 ± 0.05	0.130
Baseline higher-level revision proportion	0.17 ± 0.11	0.15 ± 0.11	0.15 ± 0.10	0.142

Values are mean ± SD unless stated otherwise. English proficiency was measured with the University English Proficiency Test (0–100). Max. |SMD| is the largest absolute pairwise standardized difference among the three conditions and provides a descriptive baseline comparison. Typing-speed and prior-AI-use summaries use available baseline values before imputation.

The study involved 297 Chinese university EFL learners who were randomly assigned in equal numbers to the control, product-feedback, and process-feedback conditions. Randomization used a NumPy-generated block sequence with block size 6 and was stratified by baseline English-proficiency band and prompt order. Allocation remained concealed until the baseline task was completed. Participants completed four argumentative writing sessions: a baseline task, two intervention tasks, and a delayed no-AI transfer task. The baseline-to-Intervention 1 and Intervention 1-to-Intervention 2 intervals were 7 days, and the Intervention 2-to-transfer interval was 14 days. The four prompts were assigned through a Latin-square structure so that prompt version was distributed across group and session. Eighteen of 297 randomized participants did not complete all sessions (6 control, 7 product-feedback, 5 process-feedback); the main reasons were schedule conflict, short illness, technical issues or incomplete tasks, and withdrawal. The final task-level dataset contained 1,153 observed writing records; 279 participants completed all four sessions.

The control condition received the same writing time, prompt sequence, and rubric information as the feedback conditions but no individualized AI feedback. The product-feedback condition received AI comments after each completed initial draft. The process-feedback condition received diagnostic comments during composing, triggered by observable pause, deletion, boundary-activity, and revision indicators. AI feedback was generated by GPT-4o (gpt-4o-2024-11-20) with temperature = 0.2 and top_p = 1.0. The configured maximum output length was 180 tokens. Product feedback was delivered once after each initial draft, whereas process feedback was limited to at most three feedback events per intervention task with a 5-minute cooldown between successive events. The feedback generator was fully automated; teachers and researchers did not edit individual AI messages before presentation. All conditions completed the transfer task without AI assistance, separating performance during feedback-supported sessions from later independent writing. The trigger rules, system configuration, and verbatim prompts are described in Supplementary Table S3 and the Supplementary material section “Feedback Prompt Specifications.”

Before the intervention, most absolute pairwise standardized differences were below 0.20 (Table 1). The largest difference was 0.253 for the recorded female proportion, followed by 0.200 for English proficiency; the remaining maxima ranged from 0.070 to 0.142. These descriptive indices summarize baseline comparability, and sample sizes were identical across conditions.

Participant retention was similar across conditions over the longitudinal sequence (Figure 1). Eighteen of 297 randomized participants did not complete all four sessions: 6 in control, 7 in product feedback, and 5 in process feedback. The main reasons were schedule conflict, short illness, technical issues or incomplete tasks, and withdrawal. In total, 93 control participants, 92 product-feedback participants, and 94 process-feedback participants completed the transfer session. Attrition was distributed similarly across conditions, while incomplete participants had lower baseline English proficiency, writing quality, and metalinguistic monitoring scores than completers (Supplementary Table S7). The longitudinal models used all observed task rows and included participant-level random effects or robust clustering to account for repeated observations from participants with partial data.

**Figure 1:**
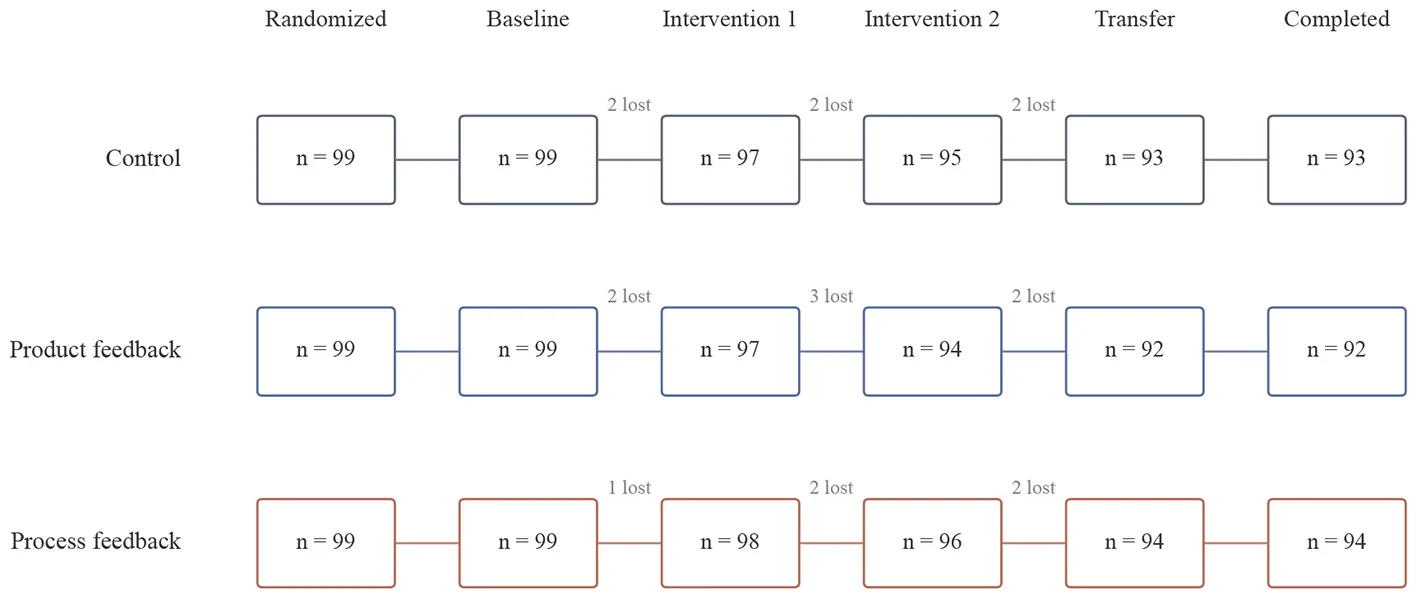
Participant flow across the baseline, intervention, and no-AI transfer sessions. The diagram reports randomized participants and retained task records by experimental condition.

### 3.2 Writing tasks and feedback conditions

Each session used an argumentative prompt designed for university EFL learners. The task sequence separated composing and revising phases so that pausing, production bursts, and revision events could be aligned with comparable phases across participants. Each task allowed 30 min of initial writing and 15 min of revision, and the target length was 250–350 words. Participants were instructed not to consult external websites, translation software, electronic dictionaries, or other generative AI tools during the study tasks. The four prompts were: (A) university students should be required to complete community service in their first year; (B) a substantial proportion of university coursework should be delivered online rather than fully face-to-face; (C) group projects should count for a larger share of university grades; and (D) universities should restrict smartphone use during face-to-face classes. Their Latin-square allocation balanced prompt exposure across conditions and sessions. This design balanced exposure but did not establish prompt equivalence; the four topics were not formally tested for difficulty before the study. Prompt fixed effects were therefore included in the statistical models to account for systematic mean differences associated with prompt version. The transfer task was completed without AI feedback in all conditions; transfer performance therefore reflected independent writing during the final task. The task requirements and complete prompt wording are consolidated in the Supplementary material section “Writing Task and Standardized Instructions.”

Product feedback was delivered after the initial draft and addressed the visible text product, including language accuracy, organization, coherence, and revision priorities. Its diagnostic input consisted of the draft text. Process feedback used ongoing behavioral indicators as diagnostic input and returned brief comments that asked the writer to inspect a process problem represented by the trigger. The process-feedback prompt instructed the model to use only observable process information, avoid inferring cognitive states, and ask an action-guiding question without providing replacement wording. The product-feedback prompt asked for brief diagnostic comments on language accuracy, organization, coherence, and revision priority without rewriting full sentences or paragraphs. The functional requirements for both prompts are summarized in the Supplementary material section “Feedback Prompt Specifications.”

Process feedback was triggered when one of four rule classes was satisfied: (1) within a 90-second window, within-clause pause proportion reached at least 0.45 and at least four pauses of 1 s or longer occurred; (2) within 60 s, at least three delete-or-replace operations occurred or at least 15 characters were deleted; (3) after at least 120 words had been produced, no sentence-boundary or paragraph-boundary pause occurred within the preceding 90 s; or (4) within 120 s, at least four modification events occurred without any revision at clause level or above. The trigger engine admitted ordinary character input, Backspace/Delete, and replacement events to the relevant trigger streams. Cursor-only movement, text selection without modification, and Undo/Redo commands did not enter trigger counts; a net change produced by Undo could remain in offline revision coding but did not add a second instance of the original deletion trigger. Paste or bulk insertion did not enter the production-burst trigger, and inactivity during a confirmed technical interruption or while the feedback window was displayed did not enter the pause trigger. Supplementary Table S3a provides the complete event-eligibility matrix. The first 5 min of each session were exempt, and when multiple rules were satisfied only the highest-priority trigger was executed. The process-feedback prompt requested approximately 35–60 English words, and messages appeared within 10 s of a trigger and remained visible until the participant closed them. The 582 process-feedback messages averaged 79.18 words (SD = 6.85, range 64–96), while the 191 product-feedback messages averaged 233.77 words (SD = 22.73, range 175–290). Every observed process-feedback intervention task received three messages, giving mean task-level totals of 237.72 words at Intervention 1 and 237.35 at Intervention 2; every product-feedback task received one message, with corresponding means of 231.08 and 236.55 words. Latency from presentation to the next substantive writing action averaged 62.33 s (SD = 26.35) for process feedback and 91.12 s (SD = 45.85) for product feedback. Average task-level word exposure was similar, while message frequency, presentation timing, and opportunities for immediate action differed. The comparison therefore concerns the two intervention packages as implemented. API timeouts, HTTP 429/5xx responses, empty responses, and format-validation failures were retried once after 2 s. A second failure generated no replacement message, was logged as generation_failed, and did not count as a successful feedback event; later qualifying triggers could still be processed. Outputs that directly rewrote a student's sentence, addressed an unrelated issue, were empty, or clearly exceeded the configured length limit were discarded and retried once. These checks handled format and explicit instruction violations; the independent message-level evaluation assessed semantic accuracy, relevance, actionability, and potentially misleading content. Feedback was delivered automatically as designed, with message quality evaluated independently after delivery.

The real-time trigger engine and the offline process analysis shared the same underlying event records but served different functions. Online triggering used simplified event-stream rules based on the current text state and cursor position to calculate indicators within fixed windows; offline analysis applied the full pause-location and revision-coding protocol to construct the final statistical variables. The online rules therefore were not treated as identical to the later linguistic coding. A 5-min cooldown separated eligible trigger events, no more than three process messages were delivered in one task, and only the highest-priority rule was executed when multiple rules were satisfied. Supplementary Table S3b provides a complete representative trigger-to-payload-to-feedback example.

The three feedback conditions separated final-text comments, online process diagnostics, and rubric-only writing practice. Product feedback tested whether AI comments on the final draft improved text quality and revision outcomes. Process feedback tested whether diagnostic prompts during composing were associated with changes in online writing behavior. The control condition held prompt order, time, and rubric exposure constant without individualized AI guidance. The condition labels describe the intervention packages as implemented, including their distinct exposure, timing, and feedback dose.

### 3.3 Feedback quality evaluation

To assess the quality of the delivered AI feedback, all 191 product-feedback messages and a stratified random sample of 191 of the 582 process-feedback messages were independently evaluated, yielding 382 rated messages. The process sample was stratified by Intervention 1 and Intervention 2. Two EFL teachers with at least three years of university English-writing teaching experience rated the messages after condition, participant, and session labels had been removed. Accuracy was coded as accurate, partly accurate, or inaccurate; actionability and relevance were rated on five-point Likert scales; and potentially misleading content was coded yes/no. The teachers first rated all messages independently, and disagreements were adjudicated by a third senior EFL teacher. Product feedback was rated accurate for 163 messages (85.3%), partly accurate for 24 (12.6%), and inaccurate for 4 (2.1%); the corresponding process-feedback counts were 169 (88.5%), 19 (9.9%), and 3 (1.6%). Potentially misleading content was identified in 8 product messages (4.2%) and 6 process messages (3.1%). Mean relevance was 4.35 (SD = 0.79) for product feedback and 4.41 (SD = 0.70) for process feedback; mean actionability was 4.29 (SD = 0.82) and 4.47 (SD = 0.66), respectively. Agreement was weighted Cohen's κ = 0.79 for accuracy, Cohen's κ = 0.76 for potentially misleading content, ICC(2,k) = 0.86 for actionability, and ICC(2,k) = 0.83 for relevance. The two message sets therefore showed high accuracy, low misleading-content rates, and high relevance and actionability under independent blind evaluation (Supplementary Table S3c).

### 3.4 Process measures and outcome variables

WriteTrace is publicly accessible through the official project website (https://writingtrace.com/); the study-specific trigger engine and feedback interface were separate integrations around the public platform. The same 24 monitoring items were administered at baseline, Intervention 1, Intervention 2, and transfer. Participants received neither the answer key nor item-level correctness feedback after any administration. Because item familiarity could contribute to repeated scores, longitudinal interpretation focuses on between-condition differences and condition-by-session change; all three conditions followed the same monitoring schedule.

The primary process dataset was recorded in the publicly available WriteTrace keystroke-logging platform (v1.2.3, build 2026-02-26). Study-specific trigger rules, feedback integration, and data-processing procedures were implemented around the platform. Tasks were completed in the English writing editor in Google Chrome 145 on Windows 11. The event record contained participant and task/session identifiers, timestamp, keydown/input/delete/replace action, character class, affected-span length, cursor position, inter-action interval, and feedback presentation and close times. Before aggregation, consecutive deletions to the same region within 2 s were merged, selection followed by replacement counted as one revision, and undo/redo was resolved according to the final net text state. Paste or bulk insertion was identified from browser input evidence, excluded from production-burst length, and coded as a revision when it altered existing text; unknown paste provenance received the source-unknown code. Cursor-only movement and selection without modification contributed no production or revision event. Confirmed technical-interruption intervals and inactivity while feedback was displayed were excluded from composing pauses, while severe interruptions with unrecoverable text state were marked as incomplete process records without keystroke imputation. Ambiguous events were coded from the text state at the time of the event, the cursor's actual location, and the final net textual change; later punctuation did not retrospectively change an earlier pause category, and inactivity was removed as a technical interruption when corroborating event evidence was present. Full rules and representative cases are described in the Supplementary material section “Process Measures and Coding Rules” and Supplementary Table S6a. Pause events were extracted from eligible inter-action intervals, and the primary threshold defined a pause as an interval of at least 1,000 ms; sensitivity analyses repeated the count models with thresholds of 1, 2, 3, and 5 s. Pause locations were coded as within-word, between-word, within-clause, sentence-boundary, or paragraph-boundary. A within-word pause occurred inside an unfinished lexical token and was followed by continued typing of the same word; a between-word pause occurred between two complete words while the same local syntactic constituent continued; and a within-clause pause occurred inside an unfinished clause, outside a within-word position, including phrase- or constituent-internal and comma-internal positions. A sentence-boundary pause occurred after a completed sentence-final boundary, with subsequent production beginning a new sentence, whereas a paragraph-boundary pause occurred at the end of a completed paragraph, with subsequent substantive production beginning a new paragraph. Larger textual boundaries took precedence when descriptions overlapped. The main pausing outcome for lower-level interruption was within-clause pause proportion. Boundary pause proportion combined sentence- and paragraph-boundary pauses because both index a higher textual location than within-clause pausing. These categories describe observable pause locations in the keystroke record (Supplementary Tables S1, S2).

Production bursts were defined as stretches of continuous text production bounded by pauses. The main burst outcome was mean burst length in words, selected to summarize the amount of continuous production within each task and to retain compatibility with the longitudinal mixed model. A task-level median-burst summary was examined as a descriptive sensitivity check and is reported in Supplementary Table S11. Revision events were coded for operation, linguistic level, function, outcome, delayed status, character-based revision distance, and whether the event followed AI feedback. Local revision referred to character-, word-, or phrase-level changes that did not alter the proposition of the clause. Higher-level revision referred to clause-, sentence-, paragraph-, or discourse-level changes, including argument relations. Successful revision fully resolved the target problem without introducing a new one; partly successful revision improved the target while leaving residual issues; ineffective revision did not improve the target; introduced-error revision created a new language or discourse problem. Effective revision combined successful and partly successful revisions. Primary revision inferences focused on higher-level and effective revision. Five thousand eight hundred forty-four of 23,374 revision events (25.0%) were double-coded by two human coders in a condition- and stage-stratified sample after 300 calibration events. The coders independently reviewed the sampled events against the manual; unresolved cases were adjudicated by a third senior researcher with expertise in EFL writing. Final agreement was κ = 0.783 for revision level and κ = 0.764 for revision effectiveness.

Metalinguistic monitoring was measured with a 24-item task following each writing session. The instrument was developed for this study from a 36-item pool covering grammar/form, lexical choice, sentence structure, cohesion/reference, organization, and argument/discourse. Three experts reviewed item relevance, clarity, category fit, and ambiguity; a 46-participant pilot then informed selection of four items per category, including two no-error items. Pilot item difficulty ranged from 0.32 to 0.78, corrected item–total correlations from 0.29 to 0.61, and KR-20 was 0.80. During the study, participants identified and located a language or discourse problem, classified its type, and rated confidence on a 1–5 scale. The main metalinguistic score was the number of correct responses from 0 to 24; confidence ratings were retained separately and did not contribute to this score. Internal consistency was KR-20 = 0.81 at baseline, 0.83 at Intervention 1, 0.85 at Intervention 2, and 0.82 at transfer. Baseline monitoring correlated with English proficiency at *r* = 0.34, *p* < 0.001. This association provides convergent evidence with proficiency while the item-level problem-identification and discourse-classification format captures a more specific evaluative skill than a general proficiency score. The development procedure, task instructions, scoring rule, complete item set, item categories, target locations, and predetermined answer classifications are described in the Supplementary material section “Metalinguistic Monitoring Measure” and Supplementary Table S4. Writing quality was measured through blinded ratings of the final text; the analysis used the mean of two raters' total scores. Post-task perceived mental effort was reported on a 1–7 scale after each task using a single item that asked how much mental effort the participant invested in the writing task; the item was administered immediately after writing and before the monitoring task. The study-specific monitoring measure has content and internal-consistency evidence, but it has not undergone extensive independent construct validation.

Table 2 links each construct to its analysis level and reliability evidence. Within-clause pause proportion was analyzed with boundary pause proportion and mean burst length, while revision frequency was paired with revision level and revision effectiveness.

**Table 2:** Operational definitions, data sources, and reliability of main variables.

Variable	Analysis level	Operational definition	Reliability or audit evidence
Writing quality	Task	Mean blinded rating of the final text on a 0–100 scale.	ICC(2,k) = 0.915.
Within-clause pause proportion	Task	Proportion of pause events occurring inside clauses among all detected pauses.	No task rows lacked pause events; invalid normalized pause times = 0.
Boundary pause proportion	Task	Proportion of pause events at sentence or paragraph boundaries.	Same pause-event audit as above.
Mean burst length	Task	Mean number of words produced between adjacent pauses.	Computed from timestamped keystroke and pause events.
Higher-level revision proportion	Task/event	Proportion of revision events at clause level or above.	Cohen's kappa = 0.783 for double-coded revision level.
Effective revision proportion	Task/event	Proportion of revisions coded as successful or partly successful.	Cohen's kappa = 0.764 for double-coded revision effectiveness.
Metalinguistic monitoring score	Task	Correct responses on 24 problem-identification and classification items.	Item-level responses available for all observed task records.
Feedback-window activity	Feedback window	Pause rate and higher-level revision rate in pre-feedback and post-feedback windows.	773 feedback events and 2,328 feedback-window records.
Feedback quality	Message	Independent blind ratings of accuracy, actionability, relevance, and potentially misleading content.	382 messages; weighted κ = 0.79 for accuracy, κ = 0.76 for misleading-content judgement, ICC(2,k) = 0.86 for actionability, and ICC(2,k) = 0.83 for relevance.

The longitudinal dataset comprised 1,153 task records from 297 randomized participants.

### 3.5 Coding, reliability, and data quality control

Two blinded university English teachers, each with three years of teaching experience, scored every final text independently with a four-dimensional analytic writing rubric yielding a total score from 0 to 100. They completed approximately 4–5 h of rubric training and calibrated their use of the rubric on 30 essays before formal scoring. The mean of their two total scores formed the writing-quality outcome, and reliability was ICC(2,k) = 0.915, estimated within the intraclass-correlation framework described by Shrout and Fleiss and by McGraw and Wong (Shrout and Fleiss, 1979; McGraw and Wong, 1996). Revision coding was checked by two human coders on 5,844 double-coded revision records, sampled in a condition- and stage-stratified design after 300 calibration events. Agreement for higher-level revision and revision effectiveness was estimated with Cohen's kappa (Cohen, 1960). The coders independently reviewed discrepant records against the operational categories; unresolved cases were adjudicated by a third senior researcher with EFL writing expertise. The operational definitions, sample-selection rule, and adjudication procedure are described in Supplementary Table S6 and the Supplementary material section “Process Measures and Coding Rules.”

The data quality audit covered participant uniqueness, duplicate task identifiers, missing process records, invalid pause times, invalid revision outcomes, feedback-session placement, writing-score range, and missing covariates. All 13 checks passed. Three participants had missing typing-speed values and 10 had missing prior-AI-use values; these small numbers of covariate omissions were median imputed to preserve the analytic sample, and missingness indicators were included in the relevant models. The median was selected as a robust location estimate for the affected covariates and avoided using model-derived values for the missing observations. The audit also confirmed that every analyzed task row had pause and revision records, and that feedback events occurred only in intervention sessions. The available-observation analyses rely on a missing-at-random working assumption conditional on observed model information; Supplementary Table S7 documents the observed baseline differences between completers and incomplete cases.

### 3.6 Statistical analysis

The analysis used all observed task records and accounted for the repeated-measures structure: tasks were nested within participants, and prompts varied across sessions. The primary continuous outcomes were modeled with mixed-effects regressions. For participant *i*, prompt *p*, and session *t*, the general specification was


$$\begin{array}{l} Y_{ipt}=\beta_{0}+\beta_{1}G_{i}+\beta_{2}S_{t}+\beta_{3}\left(G_{i}\times S_{t}\right)+\gamma^{⊤}{\text{X}}_{i}+\delta_{p}+u_{i}+\varepsilon_{ipt}, \end{array}$$ (1)

where *Y*_*ipt*_ is the outcome, *G*_*i*_ denotes feedback condition, *S*_*t*_ denotes session, *G*_*i*_ × *S*_*t*_ is the condition-by-session interaction, **X**_*i*_ contains standardized English proficiency, typing speed, and missingness indicators, δ_*p*_ is a prompt fixed effect, *u*_*i*_ is a participant random intercept, and ε_*ipt*_ is the residual. The control condition and baseline session were the reference categories, so interaction coefficients estimate condition-specific change relative to the control group's baseline-to-session change. Direct process-vs.-product contrasts were also computed explicitly. For a non-baseline session, the same-session contrast was $\left({\hat{\beta }}_{G,process}-{\hat{\beta }}_{G,product}\right)+\left({\hat{\beta }}_{GS,process,t}-{\hat{\beta }}_{GS,product,t}\right)$, whereas the difference in change from baseline used only ${\hat{\beta }}_{GS,process,t}-{\hat{\beta }}_{GS,product,t}$.

Pause counts were analyzed with a negative-binomial generalized estimating equation (GEE) to account for overdispersion and repeated observations (Liang and Zeger, 1986). The count model was


$$\begin{array}{r} \log E\left(C_{ipt}\right)=\alpha_{0}+\alpha_{1}G_{i}+\alpha_{2}S_{t}+\alpha_{3}\left(G_{i}\times S_{t}\right)+\eta^{⊤}{\text{X}}_{i}+\delta_{p}+\\ \log\left(W_{ipt}/100\right), \end{array}$$ (2)

where *C*_*ipt*_ is the number of detected pauses and *W*_*ipt*_ is final word count. The offset expresses pause counts per 100 words. Within-clause pause proportion, boundary pause proportion, and mean burst length were analyzed after logit or logarithmic transformation, respectively; their *b* coefficients therefore do not represent percentage-point changes on the original scale. Higher-level and effective revision proportions were modeled with population-averaged binomial GEE models using robust standard errors; exponentiated terms are reported as ratios. The GEE models used an exchangeable working correlation structure with participants as the clustering unit, and the main contrasts were refit under an independence working correlation as a sensitivity check. The direction and interpretation of the main contrasts were unchanged. All analyses were conducted in Python 3.12.7 with pandas 2.2.3, NumPy 2.1.3, SciPy 1.14.1, statsmodels 0.14.4, and scikit-learn 1.5.2; bootstrap intervals used 2,000 participant-level resamples.

Transfer writing quality was analyzed with an analysis-of-covariance model:


$$\begin{array}{l} Q_{iT}=\theta_{0}+\theta_{1}G_{i}+\theta_{2}Q_{i0}+\theta_{3}^{⊤}{\text{X}}_{i}+\delta_{p}+\xi_{i}, \end{array}$$ (3)

where *Q*_*iT*_ is transfer writing quality and *Q*_*i*0_ is baseline writing quality. Baseline adjustment followed the randomized-comparison logic described by (Rubin 1974). The transfer ANCOVA provided the primary no-AI transfer contrast. The direct process-vs.-product contrast was ${\hat{\theta }}_{process}-{\hat{\theta }}_{product}$, and the standardized process-feedback transfer effect was calculated as $d_{adj}=\left({\hat{\theta }}_{process}-{\hat{\theta }}_{control}\right)/{\hat{\sigma }}_{\xi }$, where ${\hat{\sigma }}_{\xi }$ is the ANCOVA residual standard deviation. A pooled raw-standard-deviation effect size was retained as a scale-based sensitivity estimate. A parallel HC3-robust linear model estimated the direct process-vs.-product contrast in transfer metalinguistic monitoring after adjustment for baseline monitoring, English proficiency, typing speed, missingness, and transfer-prompt assignment. The analysis distinguished primary outcomes from sensitivity and exploratory analyses, and estimates are reported with 95% confidence intervals. P values are two-sided nominal values without a uniform family-wise multiplicity adjustment. The sequential mediation model estimated:


$$\begin{array}{l} M_{i}=a_{1}G_{1i}+a_{2}G_{2i}+\lambda^{⊤}{\text{X}}_{i}+e_{Mi}, \end{array}$$ (4)

$$\begin{array}{l} R_{i}=dM_{i}+a_{R1}G_{1i}+a_{R2}G_{2i}+\rho^{⊤}{\text{X}}_{i}+e_{Ri}, \end{array}$$ (5)

$$\begin{array}{l} Q_{iT}=c_{1}^{\prime }G_{1i}+c_{2}^{\prime }G_{2i}+b_{M}M_{i}+b_{R}R_{i}+\psi^{⊤}{\text{X}}_{i}+e_{Qi}, \end{array}$$ (6)

where *M*_*i*_ is metalinguistic monitoring measured after Intervention 1 and *R*_*i*_ is higher-level revision measured during Intervention 2. Two orthogonal treatment contrasts represented the three conditions: *G*_1*i*_ = 0 for control, −0.5 for product feedback, and +0.5 for process feedback, making the focal coefficients direct process-vs.-product contrasts; *G*_2*i*_ = −2/3 for control and +1/3 for each AI-feedback condition, representing the combined feedback-vs.-control contrast. Monitoring, higher-level revision, transfer quality, baseline mediator values, English proficiency, and typing speed were z-standardized within the complete-case mediation sample; treatment contrasts and prompt indicators remained on their original coding scales. The reported path and indirect-effect estimates are standardized with respect to the continuous variables, and the *G*_1_ coefficients represent the full standardized process-minus-product difference. Transfer writing quality was measured 14 days after Intervention 2 in the no-AI transfer task. Indirect effects were estimated with 2,000 participant-level bootstrap samples, following regression-based mediation logic (Hayes, 2018). The mediation sample consisted of the 279 participants who completed all sessions, with baseline monitoring and baseline higher-level revision included as mediator controls. Mixed models were fitted with standard longitudinal modeling tools (Bates et al., 2015); statistical significance was evaluated with two-sided tests and 95% confidence intervals. Primary trajectory contrasts are distinguished from feedback-window, threshold-sensitivity, mediation, and state-transition analyses (Supplementary Table S2). The feedback-window intervals distinguished the proximal post-feedback period (0–1 min), an intermediate period (1–3 min), and a later within-task period (3–5 min), with the preceding 3 min serving as a local reference. The window analysis was exploratory and its cut-points were not preregistered; robustness was therefore evaluated with alternative 2–4, 3–5, and 4–6 min windows. Window estimates describe localized behavioral change around feedback events, while causal inference rests on the experimental condition structure rather than temporal proximity alone.

## 4 Results

### 4.1 Sample retention, baseline equivalence, and measurement quality

The randomized sample contained 99 participants per condition, and 279 participants completed all sessions. Eighteen of 297 randomized participants did not complete all sessions: 6 in control, 7 in product feedback, and 5 in process feedback. The main reasons were schedule conflict, short illness, technical issues or incomplete tasks, and withdrawal. The task-level analysis contained 1,153 writing records, 106,470 pause events, 23,374 revision events, and 773 feedback events. Table 1 replaces significance tests of baseline equality with pairwise standardized differences. Most maxima were below 0.20; the largest was 0.253 for the recorded female proportion, while baseline writing quality was 63.46, 64.63, and 64.35 in the control, product-feedback, and process-feedback conditions, respectively.

Measurement quality was supported by a text-rating ICC(2,k) of 0.915, Cohen's κ = 0.783 for higher-level revision, and κ = 0.764 for effective revision. The quality audit identified no duplicate participant or task identifiers, invalid revision outcomes, invalid normalized pause times, or feedback events outside intervention sessions.

The independent feedback-quality evaluation also yielded closely aligned profiles for the two intervention packages. Accurate ratings accounted for 85.3% of product messages and 88.5% of process messages; inaccurate ratings accounted for 2.1% and 1.6%, respectively. Potentially misleading content was identified in 4.2% of product messages and 3.1% of process messages. Relevance averaged 4.35 and 4.41 on the five-point scale, while actionability averaged 4.29 and 4.47 (Supplementary Table S3c). These results show that both message sets were predominantly accurate, relevant, and actionable under blind independent evaluation.

Feedback uptake was coded at the event level from the feedback text, presentation time, text snapshots, cursor records, and subsequent insertion, deletion, replacement, and revision events. Full uptake required a substantive revision addressing the principal feedback target; partial uptake indicated identifiable inspection or revision addressing part of the target. No observable uptake indicated no target-related response within the coding window. The window extended from closure of a process-feedback message to the next process message, 5 min, or the end of composing, whichever came first; for product feedback, it covered the first 5 min of the revision phase. Across 773 events, observable uptake (full or partial) occurred in 97 of 191 product-feedback events (50.8%) and 351 of 582 process-feedback events (60.3%). A second coder independently reviewed 232 events (30.0%), yielding 87.9% three-category agreement and weighted Cohen's κ = 0.82 (95% CI [0.76, 0.88]); for the binary observable-uptake classification, agreement was 93.1% with Cohen's κ = 0.86 (95% CI [0.79, 0.93]).

### 4.2 Pausing and production bursts

The first research question concerned differences in the temporal organization of pausing and production across feedback conditions. The process-feedback condition showed the clearest decline in mean task-level within-clause pause proportion from baseline to Intervention 2 (0.289 to 0.243) and maintained a lower value at transfer (0.255). The control condition changed from 0.284 at baseline to 0.277 at transfer. The complete event-weighted distribution showed the same broader pattern across all five mutually exclusive locations. In the process-feedback condition, within-clause pauses accounted for 28.7% of baseline events, 22.2% at Intervention 2, and 23.5% at transfer; sentence-boundary pauses increased from 17.7% to 22.8% and 21.8%, and paragraph-boundary pauses from 4.6% to 6.1% and 5.5%. Within-word and between-word shares changed less. Supplementary Table S2a reports all five categories by condition and session, together with overall event counts and absolute rates per 100 final-text words.

The mixed models in Table 3 aligned with these descriptive trends. On the transformed outcome scale, the process-feedback contrasts relative to control for within-clause pause proportion were negative at Intervention 1 (*b* = −0.104, 95% CI [-0.203, -0.005], *p* = 0.040), Intervention 2 (*b* = −0.214, 95% CI [-0.313, -0.115], *p* < 0.001), and transfer (*b* = −0.150, 95% CI [-0.250, -0.050], *p* = 0.003). The direct process-vs.-product contrast was reliable at Intervention 2 (*b* = −0.082, 95% CI [-0.153, -0.010], *p* = 0.025) and less precise at transfer (*b* = −0.050, 95% CI [-0.122, 0.022], *p* = 0.173). Boundary pausing showed direct process-vs.-product differences at Intervention 2 (*b* = 0.110, 95% CI [0.047, 0.174], *p* < .001) and transfer (*b* = 0.076, 95% CI [0.011, 0.141], *p* = 0.021). Thus, the most persistent location contrast involved more pausing at sentence and paragraph boundaries.

**Table 3:** Key model estimates for pausing, burst length, post-task perceived mental effort, and writing quality, based on 1,153 available task records from 297 participants.

Outcome	Contrast	Estimate	95% CI	*p* value
Within-clause pause proportion	Process feedback × Intervention 1	-0.104	[-0.203, -0.005]	0.040
Within-clause pause proportion	Process feedback × Intervention 2	-0.214	[-0.313, -0.115]	<0.001
Within-clause pause proportion	Process feedback × Transfer	-0.150	[-0.250, -0.050]	0.003
Within-clause pause proportion	Product feedback × Intervention 2	-0.098	[-0.197, 0.002]	0.055
Within-clause pause proportion	Process vs. product, Intervention 2	-0.082	[-0.153, -0.010]	0.025
Within-clause pause proportion	Process vs. product, Transfer	-0.050	[-0.122, 0.022]	0.173
Boundary pause proportion	Process feedback × Intervention 1	0.092	[0.004, 0.181]	0.041
Boundary pause proportion	Process feedback × Intervention 2	0.264	[0.176, 0.353]	<0.001
Boundary pause proportion	Process feedback × Transfer	0.154	[0.065, 0.244]	<0.001
Boundary pause proportion	Process vs. product, Intervention 2	0.110	[0.047, 0.174]	<0.001
Boundary pause proportion	Process vs. product, Transfer	0.076	[0.011, 0.141]	0.021
Mean burst length	Transfer vs. baseline, all groups	0.138	[0.057, 0.219]	<0.001
Mean burst length	Process feedback × Intervention 2	0.101	[-0.013, 0.214]	0.082
Mean burst length	Process vs. product, Intervention 2	0.069	[-0.015, 0.153]	0.107
Mean burst length	Process vs. product, Transfer	0.074	[-0.010, 0.159]	0.086
Post-task perceived mental effort	Process feedback × Intervention 1	0.404	[0.173, 0.634]	<0.001
Post-task perceived mental effort	Process feedback × Transfer	-0.191	[-0.424, 0.043]	0.109
Post-task perceived mental effort	Process vs. product, Intervention 1	0.309	[0.146, 0.472]	<0.001
Post-task perceived mental effort	Process vs. product, Intervention 2	-0.090	[-0.255, 0.076]	0.289
Post-task perceived mental effort	Process vs. product, Transfer	-0.171	[-0.339, -0.004]	0.045
Writing quality	Process feedback × Intervention 2	5.075	[3.263, 6.888]	<0.001
Writing quality	Process feedback × Transfer	3.245	[1.413, 5.077]	<0.001
Writing quality	Product feedback × Transfer	1.712	[-0.125, 3.549]	0.068
Writing quality	Process vs. product, Intervention 2	1.975	[0.442, 3.508]	0.012
Writing quality	Process vs. product, Transfer	2.136	[0.588, 3.683]	0.007

The within-clause trajectory in Figure 2 gives the clearest visual summary of the location effect. The process-feedback estimate moved downward across the intervention sessions and remained below the control condition at transfer. At Intervention 2, both the within-clause and boundary contrasts distinguished process from product feedback. At transfer, the boundary contrast remained clear, whereas the direct within-clause contrast was less precise (*p* = 0.173); the retained pattern was therefore concentrated most clearly at sentence and paragraph boundaries.

**Figure 2:**
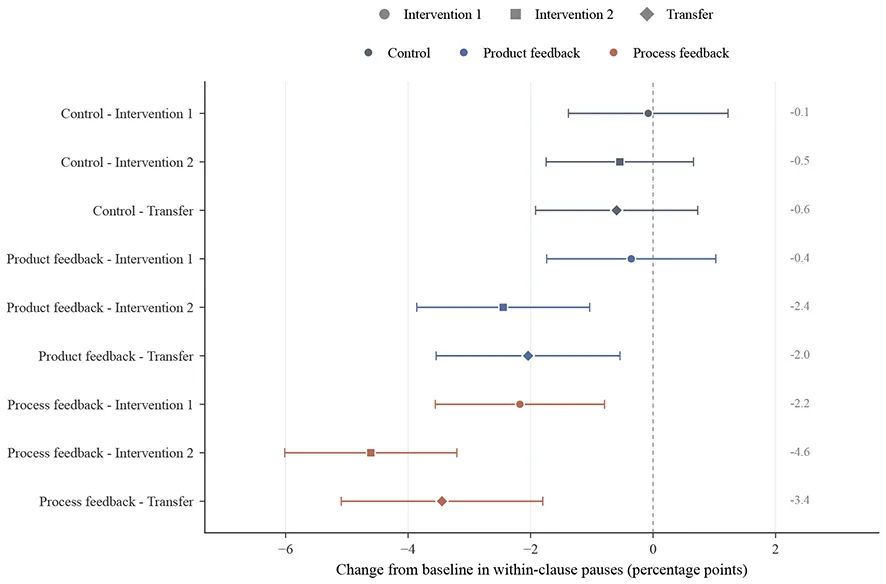
Baseline-adjusted changes in within-clause pause proportion across sessions, based on 1,153 available task records from 297 participants. Points show estimated changes from baseline by condition; intervals represent 95% confidence intervals. Lower values indicate fewer pauses occurring inside clauses.

Mean production-burst length showed a smaller and less precise trajectory (Figure 3). Process-feedback writers increased mean burst length from 5.19 words at baseline to 6.39 at Intervention 2 and 6.24 at transfer. The overall transfer effect across groups was significant, while direct process-vs.-product contrasts were *b* = 0.069 (*p* = 0.107) at Intervention 2 and *b* = 0.074 (*p* = 0.086) at transfer. A descriptive sensitivity summary based on the task-level median burst showed the same process-feedback ordering at Intervention 2 (4.464 vs. 4.324 words for product feedback) and transfer (4.404 vs. 4.163 words), with distributions reported in Supplementary Table S11. RQ1 was therefore supported most clearly by the pause-location estimates, with burst length providing a secondary directional pattern.

**Figure 3:**
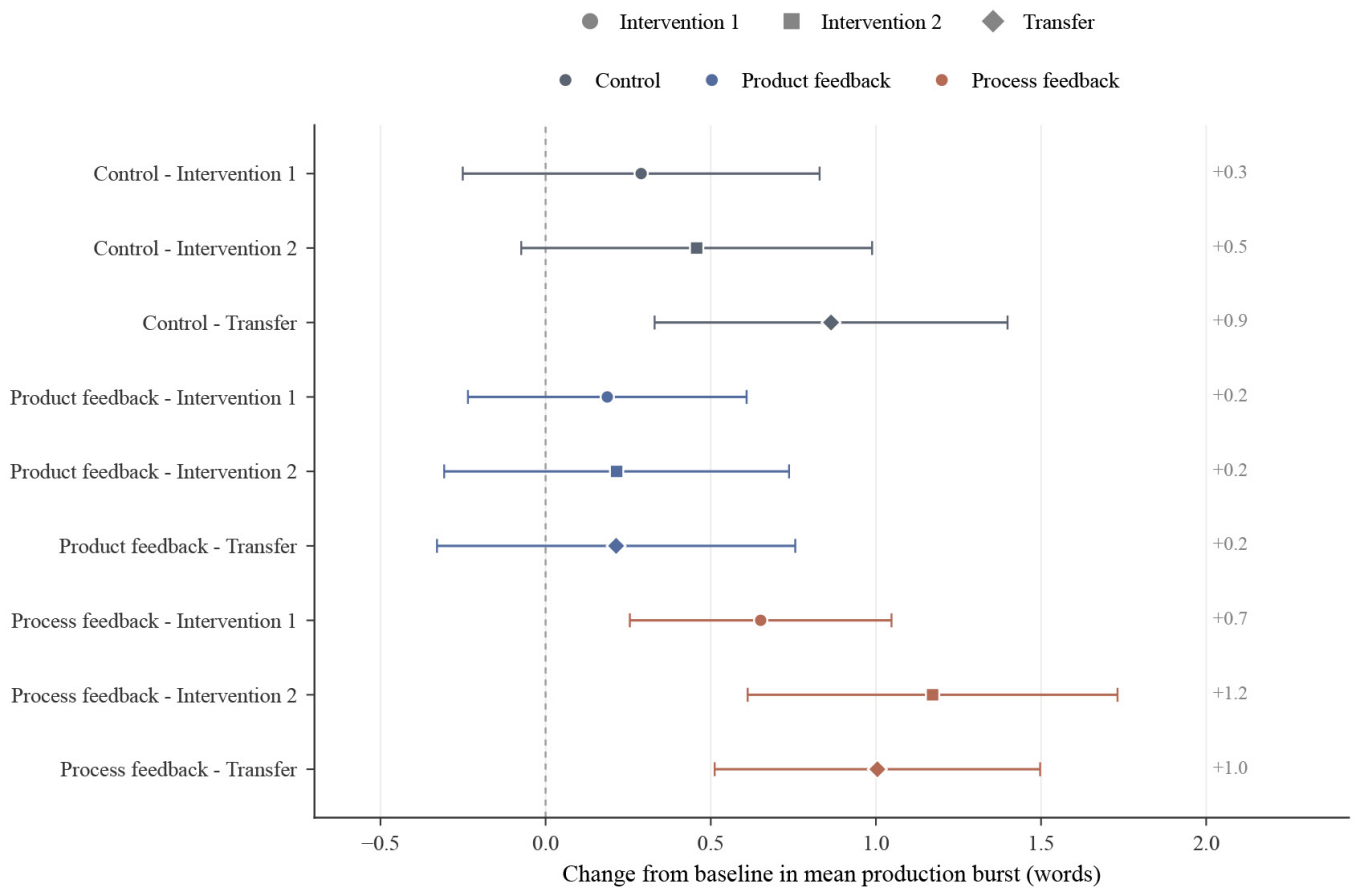
Baseline-adjusted changes in mean production-burst length across sessions, based on 1,153 available task records from 297 participants. Points show estimated changes from baseline by condition; intervals represent 95% confidence intervals. Mean burst length is expressed in words between adjacent pauses.

Negative-binomial GEE sensitivity models separated pause count from pause location. Across 1-, 2-, 3-, and 5-second thresholds, the process-feedback interaction for total pause count at Intervention 2 and transfer was not statistically robust. For example, at the one-second threshold the Intervention 2 incidence-rate ratio was 0.894 (95% CI [0.787, 1.015], *p* = 0.083); at the five-second threshold it was 1.013 (95% CI [0.872, 1.177], *p* = 0.862). The direct process-vs.-product pause-count contrasts at the one-second threshold were likewise small and non-significant at Intervention 1 (IRR = 1.014, 95% CI [0.960, 1.071], *p* = 0.618), Intervention 2 (IRR = 1.013, 95% CI [0.959, 1.071], *p* = 0.641), and transfer (IRR = 1.017, 95% CI [0.958, 1.080], *p* = 0.586). Pause location accounted for the process-feedback difference more clearly than total pauses per 100 words.

### 4.3 Revision, cognitive load, and writing quality trajectories

The second research question concerned differences in revision level and effectiveness across feedback conditions. Descriptive trends showed that both feedback conditions increased higher-level revision during intervention sessions, but process feedback retained the clearer transfer advantage. Higher-level revision proportion in the process-feedback condition rose from 0.153 at baseline to 0.282 at Intervention 2 and remained 0.243 at transfer. The product-feedback condition also increased higher-level revision at Intervention 2 (0.269) but declined to 0.203 at transfer. The control condition remained lower and changed less across sessions. The direct process-vs.-product count check showed mean total revisions of 22.90 vs. 22.60 and mean higher-level revisions of 6.46 vs. 6.07 at Intervention 2, a higher-level difference of +0.39 events per task. At transfer, the corresponding means were 20.86 vs. 20.60 for total revisions and 5.07 vs. 4.18 for higher-level revisions, a difference of +0.89 events per task (Supplementary Table S8).

Model estimates in Table 4 showed higher revision ratios for both feedback conditions relative to control. The direct process-vs.-product contrast for higher-level revision was not reliable at Intervention 2 (OR = 1.109, 95% CI [0.976, 1.259], *p* = 0.112) but was reliable at transfer (OR = 1.352, 95% CI [1.165, 1.568], *p* < 0.001). Effective revision showed a smaller direct difference: process feedback exceeded product feedback at Intervention 2 (OR = 1.195, 95% CI [1.008, 1.417], *p* = 0.040), while the transfer contrast was just above the conventional threshold (OR = 1.194, 95% CI [0.999, 1.426], *p* = 0.052).

**Table 4:** Revision and feedback-window model estimates.

Outcome	Contrast	Estimate	95% CI	*p* value
Higher-level revision ratio	Product feedback × Intervention 1	1.610	[1.247, 2.077]	<0.001
Higher-level revision ratio	Process feedback × Intervention 1	1.822	[1.431, 2.320]	<0.001
Higher-level revision ratio	Product feedback × Intervention 2	1.801	[1.427, 2.274]	<0.001
Higher-level revision ratio	Process feedback × Intervention 2	1.945	[1.546, 2.447]	<0.001
Higher-level revision ratio	Product feedback × Transfer	1.309	[1.018, 1.684]	0.036
Higher-level revision ratio	Process feedback × Transfer	1.724	[1.341, 2.215]	<0.001
Higher-level revision ratio	Process vs. product, Intervention 2	1.109	[0.976, 1.259]	0.112
Higher-level revision ratio	Process vs. product, Transfer	1.352	[1.165, 1.568]	<0.001
Effective revision ratio	Product feedback × Intervention 2	1.536	[1.193, 1.977]	<0.001
Effective revision ratio	Process feedback × Intervention 2	1.664	[1.310, 2.113]	<0.001
Effective revision ratio	Process feedback × Transfer	1.298	[1.022, 1.648]	0.032
Effective revision ratio	Process vs. product, Intervention 2	1.195	[1.008, 1.417]	0.040
Effective revision ratio	Process vs. product, Transfer	1.194	[0.999, 1.426]	0.052
Feedback-window pause rate	Post 0–1 min vs. pre-feedback	-0.092	[-0.143, -0.041]	<0.001
Feedback-window higher-level revision	Post 3–5 min vs. pre-feedback	0.026	[0.004, 0.047]	0.020

Revision models used 23,374 events from 1,153 task records; window contrasts used 582 process-feedback events.

The higher-level revision trajectory in Figure 4 separates revision level from the number of editing actions. The process-feedback curve shows a larger increase during Intervention 2 and a clearer retained increase at transfer. In the direct count analysis, the absolute higher-level event-count IRR was 1.06 at Intervention 2 (*p* = 0.251) and 1.20 at transfer (95% CI [1.08, 1.34], *p* = 0.001). After controlling total revision activity, the corresponding higher-level ratios were 1.08 at Intervention 2 (95% CI [0.95, 1.23], *p* = 0.239) and 1.30 at transfer (95% CI [1.11, 1.53], *p* = 0.001; Supplementary Table S8). Total revision activity itself showed no clear process-vs.-product difference at transfer (IRR approximately 1.02, *p* approximately 0.59). Because transfer occurred without AI support, the retained count difference shows that the higher-level revision pattern extended beyond the feedback-supported sessions.

**Figure 4:**
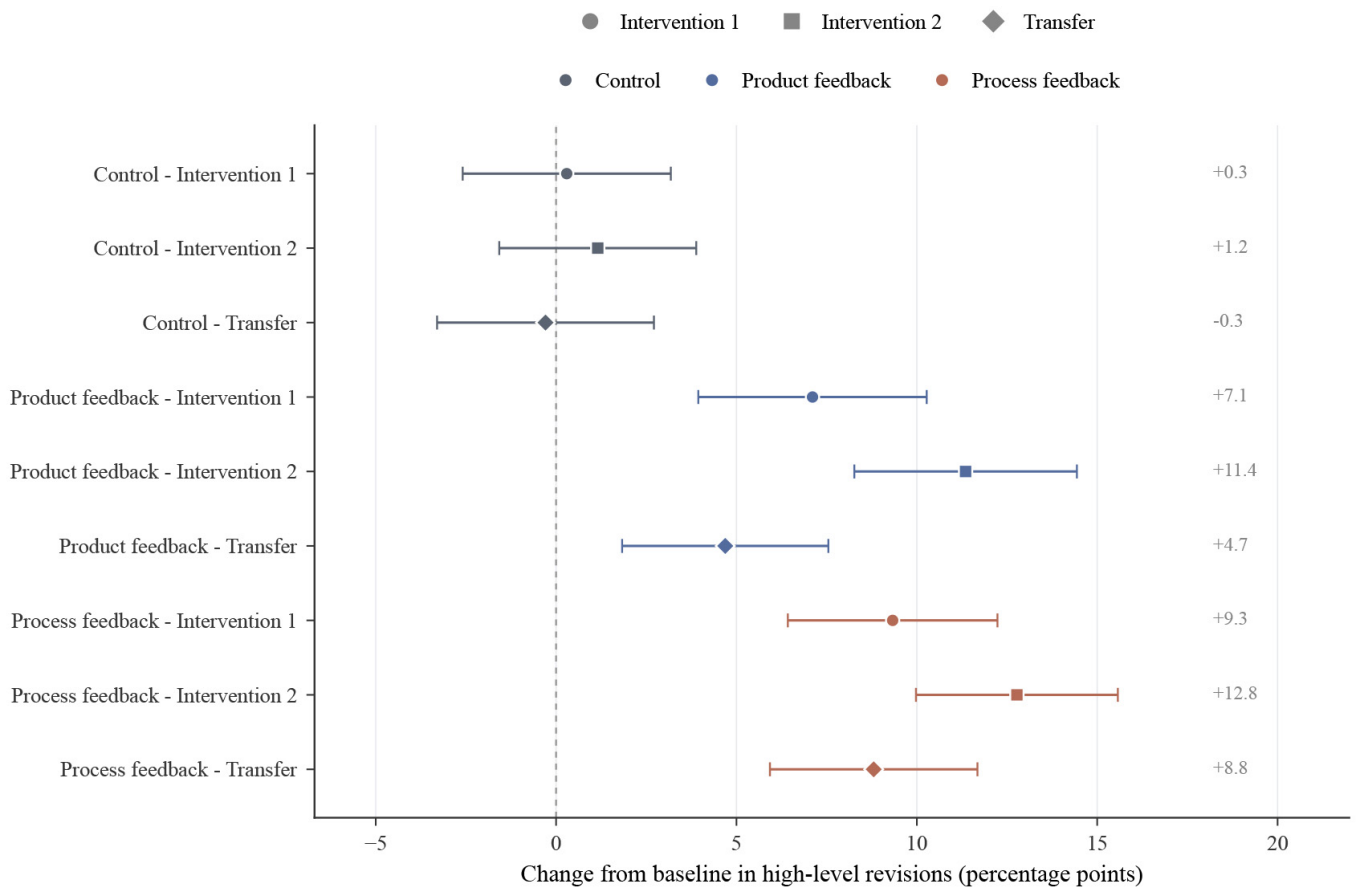
Baseline-adjusted changes in higher-level revision proportion across sessions, based on 23,374 revision events from 1,153 task records. Points show estimated changes from baseline by condition; intervals represent 95% confidence intervals. Higher-level revision refers to revision at clause level or above.

Delayed status and revision distance provided complementary evidence about where revision occurred. Process-feedback writers made more delayed revisions than product-feedback writers at Intervention 2 (31.9% vs. 27.8%; OR = 1.21, 95% CI [1.06, 1.39], *p* = 0.005) and transfer (32.8% vs. 25.6%; OR = 1.42, 95% CI [1.23, 1.64], *p* < 0.001). Their median absolute revision distance was also larger at Intervention 2 (48 vs. 41 characters; ratio = 1.13, 95% CI [1.03, 1.24], *p* = 0.009) and transfer (52 vs. 38 characters; ratio = 1.27, 95% CI [1.14, 1.41], *p* < 0.001). Introduced-error contrasts were smaller and their intervals included one. The process-feedback pattern therefore involved more frequent returns to earlier text and revisions spanning greater textual distance, alongside stable overall editing frequency at transfer (Supplementary Table S8a).

Post-task cognitive-load ratings traced an initial effort increase followed by attenuation (Figure 5). The process-feedback condition showed a higher rating at Intervention 1 relative to control (*b* = 0.404, 95% CI [0.173, 0.634], *p* < 0.001); the direct process-vs.-product difference was also positive (*b* = 0.309, 95% CI [0.146, 0.472], *p* < 0.001). The direct contrast narrowed at Intervention 2 (*b* = −0.090, 95% CI [-0.255, 0.076], *p* = 0.289) and was small and negative at transfer (*b* = −0.171, 95% CI [-0.339, -0.004], *p* = 0.045). The trajectory indicates that the higher perceived effort accompanying initial process-feedback exposure did not persist across later sessions.

**Figure 5:**
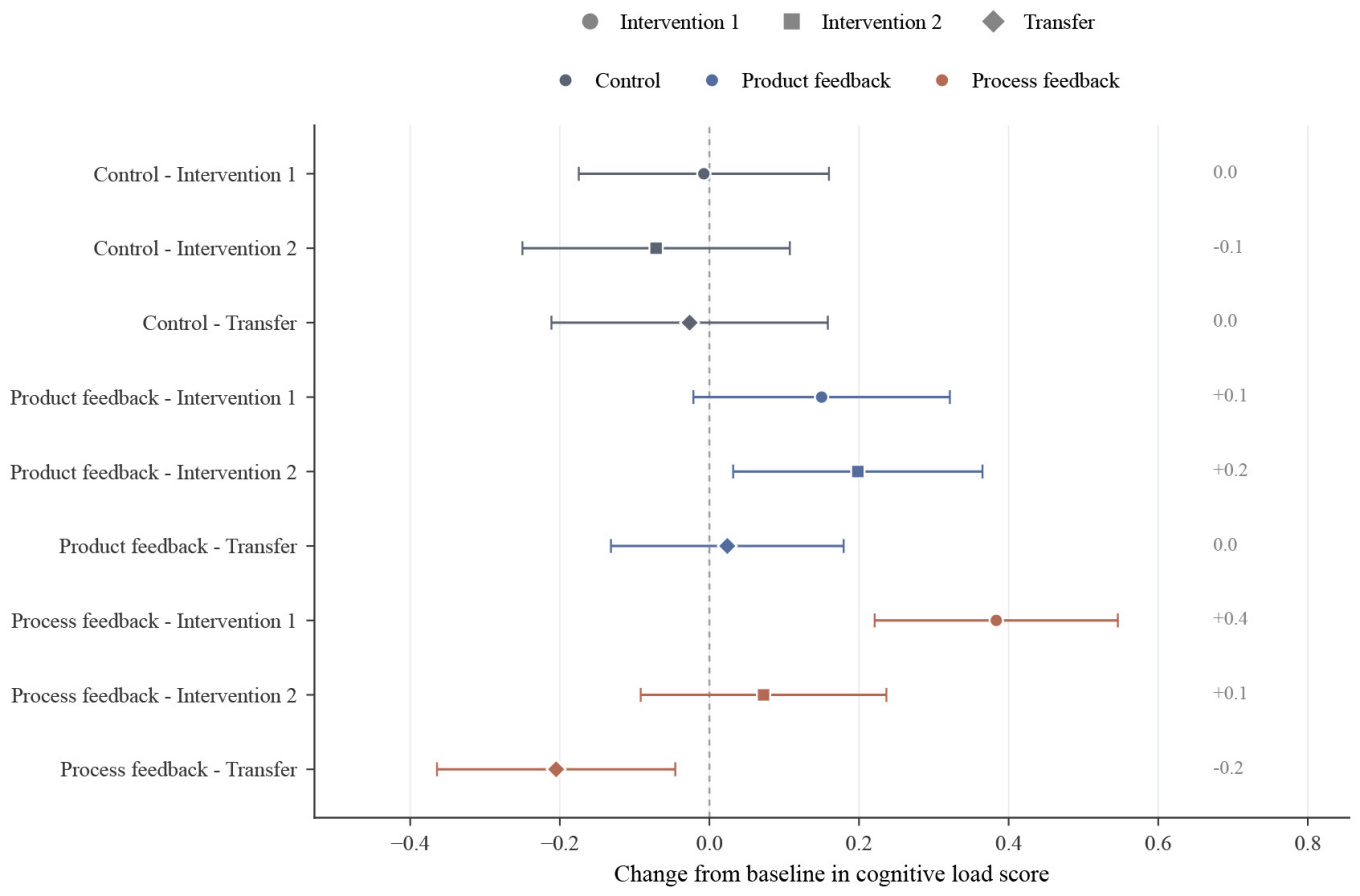
Baseline-adjusted changes in cognitive load across sessions, based on 1,153 available task records from 297 participants. Points show estimated changes from baseline by condition; intervals represent 95% confidence intervals. Cognitive load was reported on a 1–7 scale after each task.

Writing quality improved in both feedback conditions during the intervention period, with larger estimates for process feedback. In the longitudinal mixed model, process feedback showed a 5.075-point Intervention 2 advantage over the control trajectory and a 3.245-point transfer advantage. Direct process-vs.-product contrasts were also positive at Intervention 2 (Δ = 1.975, 95% CI [0.442, 3.508], *p* = 0.012) and transfer (Δ = 2.136, 95% CI [0.588, 3.683], *p* = 0.007). Because the main no-AI transfer inference used the baseline-adjusted ANCOVA, the transfer-specific estimate is reported below from that model.

Figure 6 displays the practical scale of the transfer effect. Adjusted transfer means were 67.88 for the control condition, 69.22 for product feedback, and 71.17 for process feedback. The process-feedback vs. control contrast was 3.291 points (95% CI [1.707, 4.874], *p* < 0.001), whereas the product-feedback vs. control contrast was 1.340 points (95% CI [-0.242, 2.921], *p* = 0.097). The direct process-vs.-product ANCOVA contrast was 1.951 points (95% CI [0.414, 3.489], *p* = 0.013). Standardized by the ANCOVA residual standard deviation, the process-feedback vs. control transfer effect was *d* = 0.634 (bootstrap 95% CI [0.352, 0.972]); the pooled raw-standard-deviation sensitivity estimate was *d* = 0.399. The corresponding direct process-vs.-product standardized contrast was *d* = 0.376 (bootstrap 95% CI [0.096, 0.680]), with a pooled raw-standard-deviation sensitivity estimate of *d* = 0.239.

**Figure 6:**
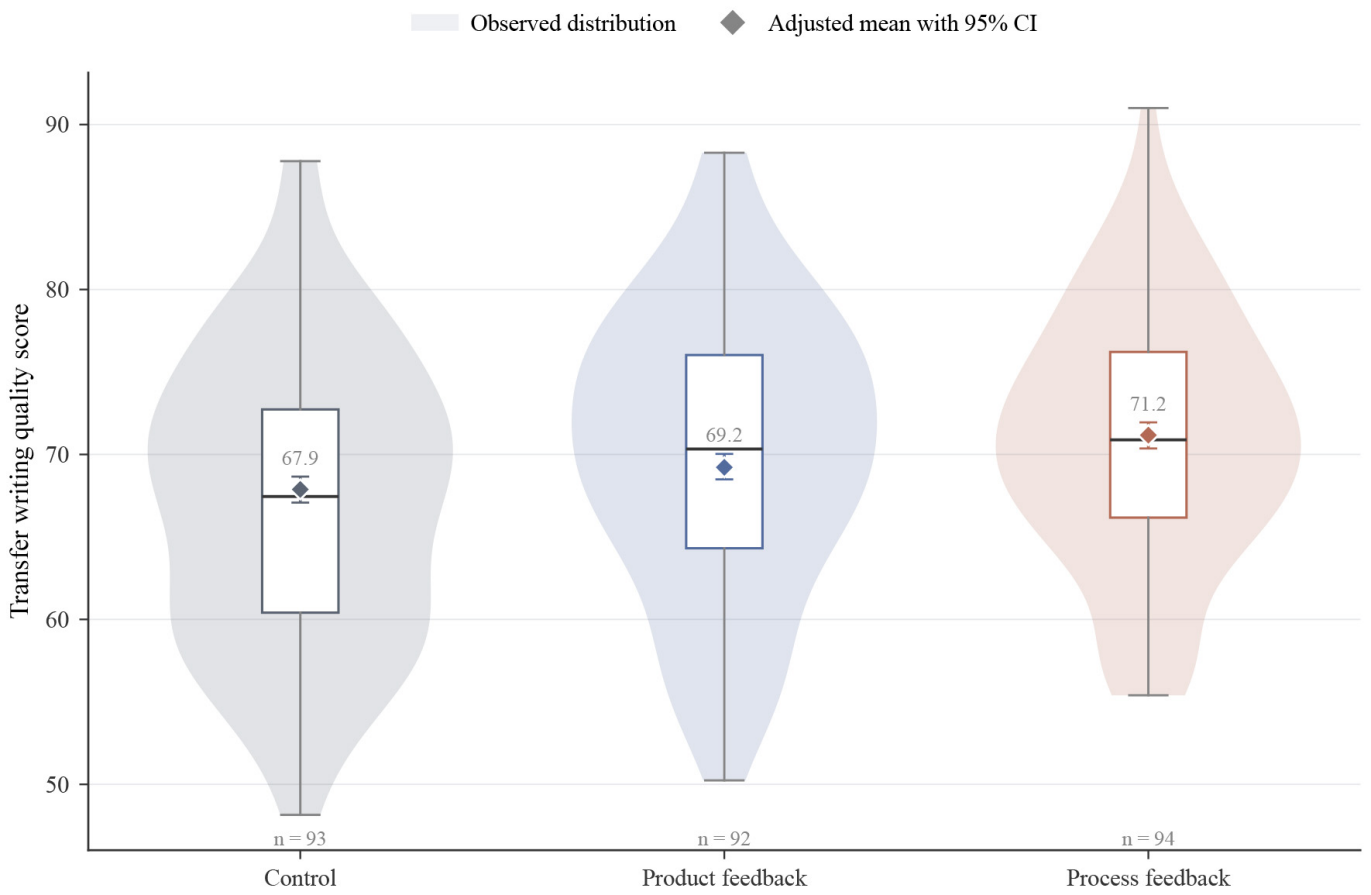
Transfer writing quality by condition (N=279: 93 control, 92 product feedback, and 94 process feedback). The distributional display is paired with baseline-adjusted means and 95% confidence intervals from the transfer ANCOVA. Transfer was completed without AI feedback.

### 4.4 Feedback windows, mediation, and state transitions

The exploratory feedback-window analysis asked how writing activity was organized immediately after a diagnostic prompt. Each of the 582 process-feedback events contributed a pre-feedback record and three post-feedback records, yielding 2,328 window-level observations. During Intervention 2, the mean higher-level revision rate rose from 0.122 (participant-cluster bootstrap 95% CI [0.109, 0.135]) before feedback to 0.182 [0.164, 0.200] in the 3–5 min post-feedback window. The 3–5 minute estimate was positive (*b* = 0.026, 95% CI [0.004, 0.047], *p* = 0.020), while the immediate 0–1 minute window showed a negative pause-rate estimate. Higher-level revision therefore emerged most clearly several minutes after feedback presentation.

The interval plot in Figure 7 locates the highest higher-level revision rate in the 3–5 min window during Intervention 2 and displays uncertainty around every estimate (Supplementary Table S9). An alternative set of 4-min windows produced the same ordering, with Intervention 2 rates of 0.119 [0.097, 0.141] before feedback, 0.141 [0.113, 0.170] at 0–2 min, 0.163 [0.128, 0.200] at 2–4 min, and 0.191 [0.151, 0.231] at 4–6 min. The aligned trajectories across window specifications strengthen the descriptive evidence for delayed revision activity after feedback.

**Figure 7:**
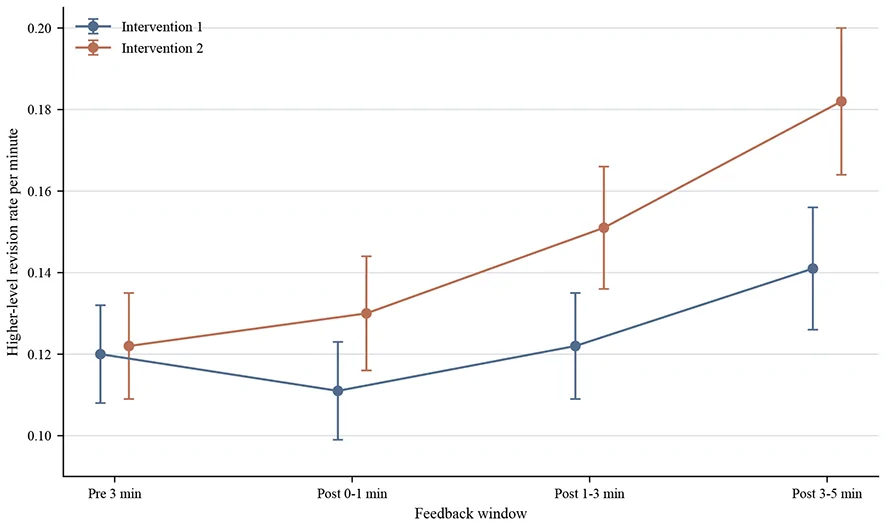
Feedback-window higher-level revision rates across 582 process-feedback events and 2,328 event-window records. Points show event-window means and vertical bars show 95% participant-cluster bootstrap confidence intervals; estimates are provided for the pre-feedback, 0–1, 1–3, and 3–5 minute windows in Intervention 1 and Intervention 2.

A matched pseudo-trigger analysis separated the post-feedback pattern from comparable changes around periods without feedback. The analysis matched 566 actual-feedback events on session, trigger class, elapsed time, and pre-trigger activity. The original Intervention 2 higher-level-revision estimate was +0.026 (95% CI [0.004, 0.047], *p* = 0.020); the matched pseudo-trigger change was +0.013, producing an adjusted feedback contrast of +0.016 (95% CI [0.003, 0.029], *p* = 0.016). The corresponding adjusted immediate pause contrast was −0.058 (95% CI [−0.098, −0.018], *p* = 0.005). Alternative windows centered on 2–4, 3–5, and 4–6 minutes yielded higher-level-revision effects from +0.012 to +0.016. These estimates consistently locate an immediate pause reduction followed by increased higher-level revision, within the behavior-triggered window design.

Table 5 reports the transfer, mediation, and pause-count robustness results. In the transfer ANCOVA, the adjusted process-feedback mean exceeded the product-feedback mean by 1.951 points after baseline quality and covariates were included, while the residual-standard-deviation effect-size estimate placed the process-feedback vs. control contrast at *d* = 0.634. A separate baseline-adjusted model estimated a 2.442-point process-vs.-product difference in transfer monitoring (95% CI [1.784, 3.100], *p* < .001). In the mediation analysis, process feedback was associated with higher monitoring (*a* = 0.737, 95% CI [0.595, 0.883]), and monitoring was associated with transfer quality (*b* = 0.305, 95% CI [0.200, 0.414]). The indirect association through monitoring was 0.225 (95% CI [0.139, 0.319], *p* < .001). The indirect association through higher-level revision alone and the full sequential pathway were smaller, with intervals spanning zero. The mediation structure therefore concentrated the indirect statistical association in metalinguistic monitoring.

**Table 5:** Transfer, mediation, and pause-count robustness results.

Result	Estimate	95% CI	*p* value	Interpretation
Adjusted transfer mean, control	67.88	[67.08, 68.66]	–	Reference condition.
Adjusted transfer mean, product feedback	69.22	[68.49, 70.03]	–	Intermediate adjusted mean.
Adjusted transfer mean, process feedback	71.17	[70.36, 71.95]	–	Largest adjusted mean.
Product feedback vs. control	1.340	[-0.242, 2.921]	0.097	Smaller transfer contrast.
Process feedback vs. control	3.291	[1.707, 4.874]	<0.001	Adjusted transfer contrast.
Process feedback vs. product feedback	1.951	[0.414, 3.489]	0.013	Direct transfer contrast.
Standardized process vs. control transfer effect	*d* = 0.634	[0.352, 0.972]	–	ANCOVA residual SD denominator.
Standardized process vs. product transfer effect	*d* = 0.376	[0.096, 0.680]	–	Direct standardized contrast.
Transfer monitoring, process vs. product	2.442	[1.784, 3.100]	<0.001	Baseline-adjusted score-point contrast.
Process feedback → metalinguistic monitoring	0.737	[0.595, 0.883]	<0.001	Interval excludes zero.
Metalinguistic monitoring → transfer quality	0.305	[0.200, 0.414]	<0.001	Positive adjusted association.
Indirect effect through monitoring	0.225	[0.139, 0.319]	<0.001	Bootstrap interval excludes zero.
Indirect effect through revision	0.018	[-0.012, 0.054]	0.238	Not reliably different from zero.
Sequential indirect effect	0.003	[-0.003, 0.013]	0.398	Interval includes zero.
Total indirect effect	0.246	[0.154, 0.344]	<0.001	Bootstrap interval excludes zero.
Pause count sensitivity, process feedback at Intervention 2	IRR 0.894–1.013	Thresholds 1–5 s	all *p* > 0.08	Total pause count was not robust.
Pause count sensitivity, process feedback at transfer	IRR 0.951–1.030	Thresholds 1–5 s	all *p* > 0.43	Location was more informative than count.
Pause count, process vs. product, Intervention 1	1.014	[0.960, 1.071]	0.618	Direct 1-s-threshold contrast.
Pause count, process vs. product, Intervention 2	1.013	[0.959, 1.071]	0.641	Direct 1-s-threshold contrast.
Pause count, process vs. product, Transfer	1.017	[0.958, 1.080]	0.586	Direct 1-s-threshold contrast.

Transfer and mediation analyses used N=279; pause-count sensitivity models used 1,153 available task records.

Figure 8 separates the reliable and imprecise mediation paths. Process feedback had a strong path to metalinguistic monitoring and a direct path to higher-level revision, but the link from metalinguistic monitoring to higher-level revision was imprecise, and the revision-to-quality path was also imprecise. The bootstrap results place the indirect transfer association more clearly in metalinguistic monitoring than in the full sequential pathway through higher-level revision.

**Figure 8:**
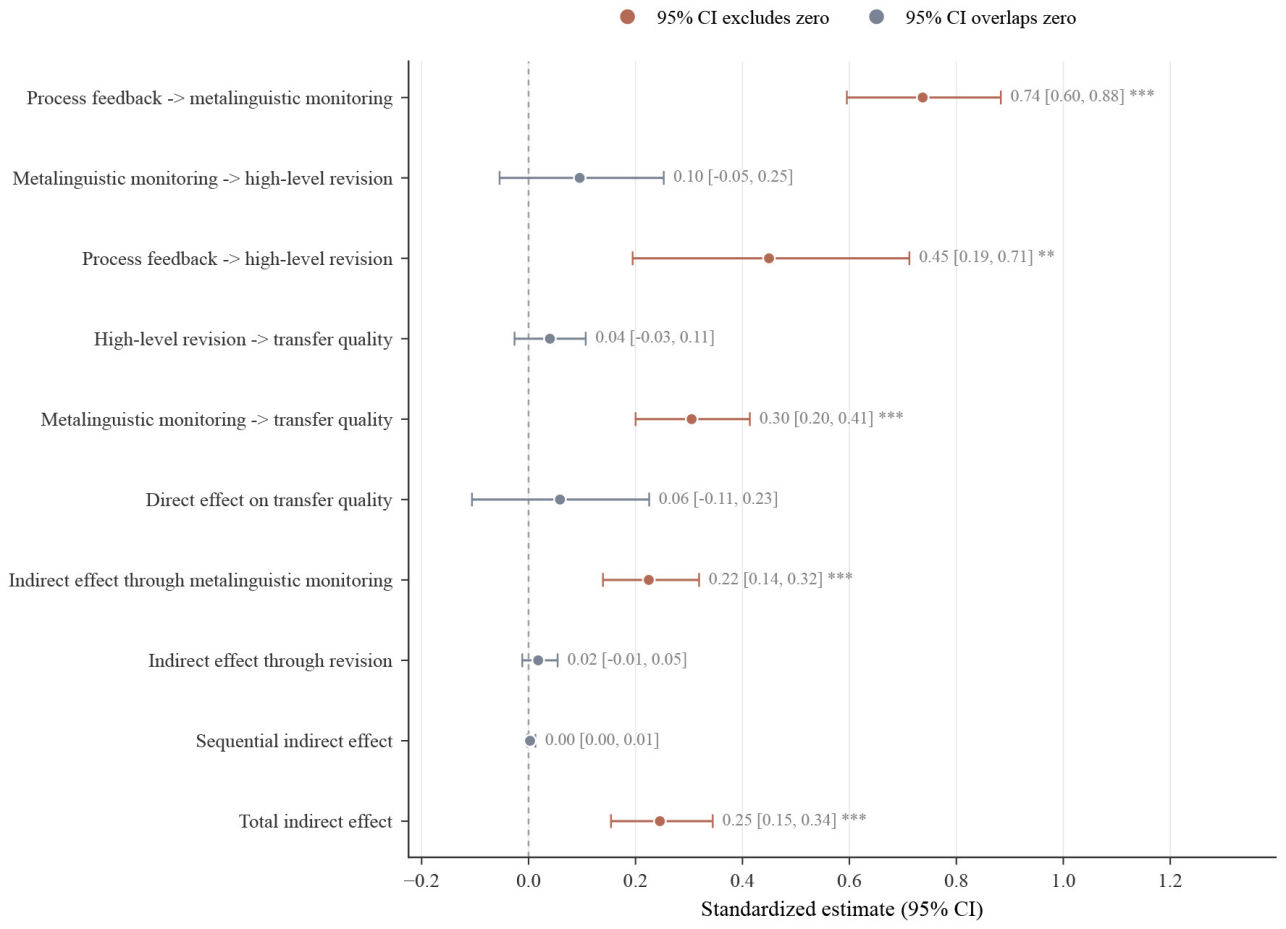
Sequential mediation estimates for process feedback, metalinguistic monitoring, higher-level revision, and transfer writing quality (N=279). Points show standardized path or effect estimates; intervals represent bootstrap 95% confidence intervals. **p < 0.01; ***p < 0.001.

The exploratory state-transition analysis characterized how writing activity was dynamically organized across planning, production, monitoring, local revision, and global revision states. Here, global revision used the same clause-, sentence-, paragraph-, and discourse-level boundary as higher-level revision. At Intervention 2, process-feedback writers were more likely than control writers to move from local revision to global revision (+0.118, 95% CI [0.052, 0.181], BH-FDR *q* = 0.008) and less likely to remain in production (-0.072, 95% CI [-0.116, -0.030], *q* = 0.013). Greater persistence in global revision was also visible descriptively (+0.082, 95% CI [0.010, 0.151]), although this contrast did not remain supported after correction (*q* = 0.141). Figure 9 and Supplementary Table S10 report all 25 participant-bootstrap intervals and adjusted q values. The two FDR-supported differences indicate less sustained text production and more movement from local editing into clause-level-or-larger revision in the process-feedback condition.

**Figure 9:**
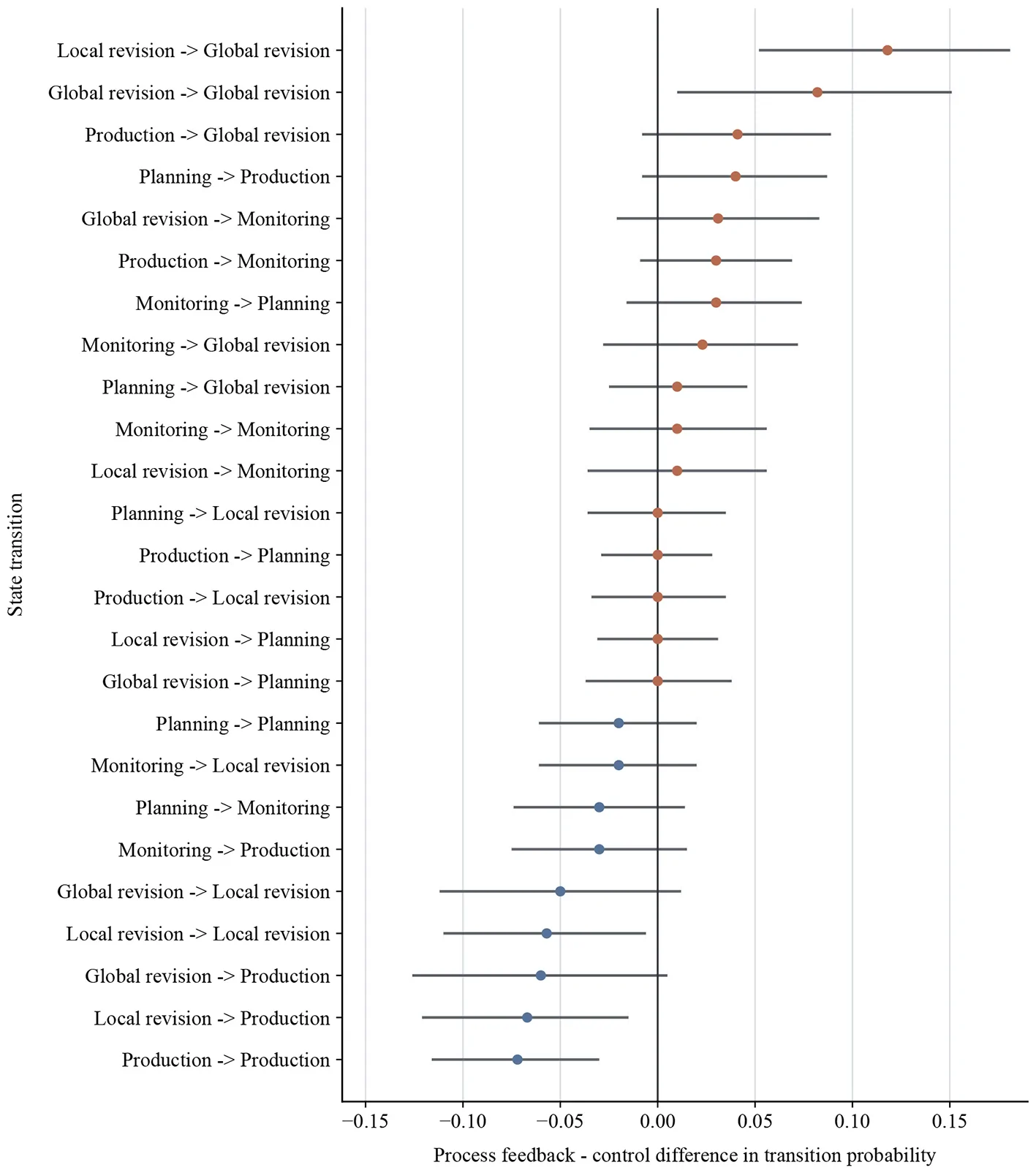
Process-feedback minus control differences in Intervention 2 state-transition probabilities. Points show probability differences and horizontal bars show 95% participant-level bootstrap confidence intervals; the analysis included 96 process-feedback and 95 control participants and transitions across 20 time bins. Positive values indicate transitions more likely in the process-feedback condition.

## 5 Discussion

Process feedback was associated with a distinct organization of writing activity across the longitudinal sequence. The clearest contrast involved pause location: process-feedback writers showed fewer within-clause pauses and more sentence- and paragraph-boundary pauses at the second intervention, and the boundary-pause difference remained visible in the no-AI transfer task. Higher-level revision also remained more frequent at transfer, accompanied by a higher adjusted transfer score. Together, these patterns connect online diagnostic prompts with the coordination of production and review described in cognitive models of writing (Flower and Hayes, 1981; Hayes, 2012).

Pause location provides the strongest behavioral account of this coordination. Across the five-category distribution, the process-feedback trajectory was characterized by lower within-clause shares and higher sentence- and paragraph-boundary shares, with the transfer contrast clearest for boundary pausing. Boundary locations give writers an opportunity to coordinate propositions, sentence relations, and paragraph development after a textual unit has been completed (Medimorec and Risko, 2017; Révész et al., 2017). The repeated contrast suggests that process-feedback writers increasingly organized interruptions around these larger units. Mean production bursts also lengthened descriptively by the second intervention and at transfer, though their direct process-vs.-product intervals remained wider. The combined pattern therefore centers on the location of interruption, with continuity of production providing supporting evidence.

Revision outcomes show a related shift in the textual level of writers' activity. Both feedback conditions increased higher-level revision during the intervention sessions, but the process-feedback advantage was clearest after feedback had been removed. At transfer, process and product writers made nearly identical numbers of revisions overall, 20.86 and 20.60 per task, yet process-feedback writers made 5.07 higher-level revisions compared with 4.18 in the product condition. The absolute higher-level IRR was 1.20 (95% CI [1.08, 1.34], *p* = 0.001), and the adjusted ratio remained 1.30 (95% CI [1.11, 1.53], *p* = 0.001) after controlling total revision activity.

The feedback-window and state-transition analyses further characterized how revision unfolded. Higher-level revision peaked 3–5 minutes after process feedback during Intervention 2, and the matched pseudo-trigger comparison retained an adjusted contrast of +0.016 (95% CI [0.003, 0.029], *p* = 0.016). Alternative windows produced closely aligned estimates from +0.012 to +0.016, indicating that the localized revision pattern did not depend on one set of cut-points. Within the transition family, process-feedback writers showed less persistence in production and more movement from local to global revision after BH-FDR correction. These temporal results are consistent with diagnostic prompts being followed by a period of inspection that develops into broader revision over several minutes; the estimates describe behavioral organization around feedback events, while the experimental condition structure provides the basis for intervention-level inference.

Metalinguistic monitoring formed the clearest statistical pathway to later performance. Process feedback was associated with higher monitoring after Intervention 1, monitoring was associated with transfer quality, and the indirect association through monitoring was 0.225 (95% CI [0.139, 0.319], *p* < 0.001). The revision-only indirect effect and the full sequential pathway had intervals spanning zero. This structure separates identifying and evaluating a language or discourse problem from carrying out a higher-level revision, a distinction central to feedback uptake (Hyland and Hyland, 2006; Ferris, 2010). The model locates the strongest indirect signal in metalinguistic monitoring; as specified in Methods, it represents a statistical pathway among observed variables.

Transfer performance gives this process pattern practical weight. In the no-AI task, the adjusted process-feedback mean exceeded the product-feedback mean by 1.951 points (95% CI [0.414, 3.489], *p* = 0.013); the corresponding standardized contrast was *d* = 0.376 (bootstrap 95% CI [0.096, 0.680]). The process-vs.-control contrast was 3.291 points, with *d* = 0.634 based on the ANCOVA residual standard deviation. These differences appeared alongside retained boundary pausing, higher-level revision, and metalinguistic monitoring. Earlier AWE and generative-AI studies have documented gains in writing performance and feedback engagement (Li et al., 2015; Li, 2024; Yeung, 2025; Luo and Zhan, 2026). The present findings add evidence that the timing and diagnostic architecture of feedback are associated with how writers organize activity when AI support is no longer present.

Post-task perceived-mental-effort ratings clarify how that organization developed. The first process-feedback exposure produced higher perceived mental effort than the control and product conditions, while the direct process-vs.-product contrast attenuated at Intervention 2 and became small and negative at transfer. This trajectory is consistent with an initial cost of integrating feedback into ongoing composition, followed by adaptation across repeated sessions. Process feedback interrupts production and requires writers to inspect an active problem, so early effort is expected when the routine is unfamiliar. The rating is a retrospective, single-item report of perceived task demand; it provides an experiential measure of effort accompanying the task rather than a direct observation of moment-to-moment cognitive processing. The later combination of lower reported effort, boundary-oriented pausing, and stronger transfer performance indicates that the initial demand did not persist at the same level.

The comparison also highlights the instructional architecture of process feedback. Product messages were longer and delivered once after drafting; process messages were shorter, repeated, and embedded in composing, while total task-level word exposure was similar. Independent blind evaluation showed high accurate ratings in both conditions (85.3% product; 88.5% process), low misleading-content rates (4.2%; 3.1%), and mean relevance above 4.3 on the five-point scale. Process messages also received a somewhat higher mean actionability rating (4.47 vs. 4.29). Feedback was delivered automatically as designed, with semantic and instructional quality evaluated independently rather than altered through teacher mediation before delivery. For instruction, the findings support prompts that direct attention to a detected composing pattern, specify an object for inspection, and leave the revision decision with the learner. Teacher-guided reflection and subsequent no-AI revision provide testable ways to extend this design and strengthen independent evaluation of diagnostic information (Pretorius and Thewissen, 2026; Lee and Mao, 2024).

The evidence is bounded by four controlled writing sessions with Chinese university EFL learners and one no-AI transfer task. Latin-square allocation balanced prompt exposure, with prompt fixed effects included because the four topics were not formally equated for difficulty. Available-observation models incorporated incomplete longitudinal records under a missing-at-random working assumption; incomplete participants began with lower proficiency, writing quality, and monitoring scores. The study-specific monitoring instrument has content and internal-consistency evidence, alongside a moderate proficiency association, but requires validation against independent metalinguistic-knowledge and learning measures. The two feedback packages differed in timing, frequency, message length, and opportunities for immediate action, and the process messages exceeded the requested 35–60-word range. Independent message ratings characterized the accuracy, relevance, actionability, and potentially misleading content of both feedback packages. The post-task perceived-mental-effort rating was retrospective and coarse, and the feedback-window estimates were organized around behavior-triggered events. These boundaries establish a focused research agenda: longer intervention sequences, multiple transfer tasks, matched feedback exposure, alternative trigger schedules, and combinations of keystrokes with screen capture, eye tracking, stimulated recall, and teacher mediation. Such designs can test the durability of the transfer pattern and specify how diagnostic prompts interact with attention and instructional support, alongside mixed findings from unrestricted AI-supported practice in other educational settings (Svendsen et al., 2024; Bastani et al., 2025).

## 6 Conclusion

AI process feedback was associated with a distinct organization of L2 writing activity across intervention and transfer. The pause-location profile combined lower within-clause pausing during the intervention with higher sentence- and paragraph-boundary pausing, and the boundary contrast remained the clearer direct difference at transfer. Process-feedback writers also returned more often to earlier text, revised across greater character distances, and retained more higher-level revision than product-feedback writers during no-AI transfer; the adjusted transfer-quality difference was 1.951 points. Metalinguistic monitoring formed the clearest indirect statistical pathway, while the revision-only and sequential pathways were imprecise. These findings position process-oriented prompts as diagnostic supports associated with independent monitoring, broader revision, and later text quality.

## Data Availability

The raw data supporting the conclusions of this article will be made available by the authors, without undue reservation.
