# Supplementary material for: From product scoring to online language processing: associations of AI-supported process feedback with pausing and revision trajectories in L2 writing
Source: Front Psychol. 2026 Oct 21;17:1951427. doi: 10.3389/fpsyg.2026.1951427 (PMC13731409; doi:10.3389/fpsyg.2026.1951427)
Supplement: Supplementary file 1 [file Data_Sheet_1.pdf]

Supplementary Material

1 OPERATIONAL DEFINITIONS AND ANALYSIS LEVELS

Table S1. Operational definitions and analysis levels of the principal variables.

Variable	Analysis level	Operational definition	Behavioural or textual feature represented
Within-clause pause proportion	Task	Pause events coded inside an unfinished clause, outside a within-word position and including phrase/constituent-internal and comma-internal positions, divided by all detected pause events.	Captures the distribution of pauses within unfinished clauses during composing.
Boundary pause proportion	Task	Sentence-boundary and paragraph-boundary pause events combined and divided by all detected pause events.	Captures the distribution of pauses at sentence and paragraph boundaries.
Mean production-burst length	Task	Mean number of words produced between adjacent pause events.	Captures the continuity of text production between qualifying pauses.
Higher-level revision proportion	Event/task	Revision events at clause level or above divided by all coded revision events.	Clause-level or larger revision.
Effective revision proportion	Event/task	Successful and partly successful revision events divided by all coded revision events.	Combines successful and partly successful revision outcomes.
Metalinguistic monitoring score	Task	Number of correct responses on the 24-item monitoring task, scored from 0 to 24.	Captures accuracy in identifying, locating, and classifying language and discourse problems.
Cognitive-load rating	Task	Single-item report obtained after each writing task on a 1–7 scale.	Captures retrospective perceived mental effort reported immediately after the writing task.
Transfer writing quality	Task	Mean of two blinded ratings on the final no-AI transfer text.	Captures rubric-based quality in the delayed independent writing task.

2 STATISTICAL MODEL SPECIFICATIONS

Table S2. Model families, coefficient scales, and analysis roles.

Outcome or analysis	Model family	Reported scale and repeated-measures structure	Analysis role and notes
Continuous task outcomes	Linear mixed-effects regression	Participant random intercept; prompt fixed effect; condition by session interactions. All mixed models converged without singular-fit warnings, and residual-versus-fitted and Q-Q plots were inspected.	Primary trajectory analyses.
Pause and boundary proportions	Mixed-effects regression on transformed proportions	Coefficients are reported on the transformed scale; the primary pause threshold is $\geq 1{,}000$ ms, with 1-, 2-, 3-, and 5-second sensitivity thresholds.	Primary process outcomes.
Mean burst length	Mixed-effects regression on a logarithmic outcome	Coefficients are on the transformed scale unless back-transformed estimates are supplied.	Secondary process outcome for production continuity.
Pause counts	Negative-binomial GEE with word-count offset	Population-averaged incidence-rate ratios; exchangeable working correlation with robust sandwich standard errors and an independence refit for sensitivity.	Sensitivity outcome separating count from location.
Higher-level and effective revision	Binomial GEE with robust standard errors	Exponentiated coefficients are reported as ratios; 5,844 of 23,374 events were double-coded in a condition- and stage-stratified sample.	Primary revision outcomes.
Feedback windows	GEE for window-level rates	Pre-feedback and post-feedback windows were clustered around process-feedback events.	Window-level analysis of activity surrounding feedback presentation.
Transfer quality	Baseline-adjusted ANCOVA with robust standard errors	Group contrasts are in score points on the 0–100 rubric scale. The primary standardized effect uses the ANCOVA residual SD; a pooled raw-SD effect is a sensitivity estimate.	Transfer outcome.
Transfer monitoring	Baseline-adjusted linear model with HC3 robust standard errors	Direct process-versus-product contrast in score points on the 0–24 scale, adjusted for baseline monitoring and analysis covariates.	Secondary transfer-process contrast.
Mediation	Regression-based path model with 2,000 bootstrap resamples	$G_1 = (0, -0.5, +0.5)$ for control, product, and process feedback provides the direct process-versus-product contrast; orthogonal $G_2 = (-2/3, +1/3, +1/3)$ represents both AI-feedback conditions versus control. Continuous model variables were z-standardized within the complete-case sample; contrasts and prompt indicators were not standardized. M_i was measured after Intervention 1, R_i during Intervention 2, and Q_{iT} at transfer.	Secondary statistical pathway analysis with participant-level bootstrap resampling.
State transitions	Aggregate transition-probability summaries	Probability differences summarize transitions across 20 time bins.	Exploratory dynamic process analysis.

Table S2a. Distribution of pause locations at the 1,000-ms threshold.

Session	Condition	Within-word	Between-word	Within-clause	Sentence boundary	Paragraph boundary
Baseline	Control	17.6%	31.8%	28.0%	18.0%	4.6%
Baseline	Product	17.4%	32.2%	27.9%	18.0%	4.5%
Baseline	Process	17.8%	31.2%	28.7%	17.7%	4.6%
Intervention 1	Control	17.3%	32.1%	27.8%	18.1%	4.7%
Intervention 1	Product	17.0%	32.6%	25.8%	19.5%	5.1%
Intervention 1	Process	16.8%	32.5%	25.0%	20.4%	5.3%
Intervention 2	Control	17.2%	32.6%	27.4%	18.1%	4.7%
Intervention 2	Product	16.9%	32.8%	24.7%	20.3%	5.3%
Intervention 2	Process	16.4%	32.5%	22.2%	22.8%	6.1%
Transfer	Control	17.4%	33.0%	27.2%	17.9%	4.5%
Transfer	Product	17.1%	33.2%	25.1%	19.8%	4.8%
Transfer	Process	16.6%	32.6%	23.5%	21.8%	5.5%

Location	Events	Percentage of all pauses	Events per 100 final-text words
Within-word	18,421	17.30%	5.29
Between-word	35,029	32.90%	10.07
Within-clause	28,547	26.81%	8.21
Sentence boundary	19,585	18.39%	5.63
Paragraph boundary	4,888	4.59%	1.40
Total	106,470	100.00%	30.60

Session-by-condition percentages are event-weighted distributions within each cell. For category k, $P_k = N_k/N_{\text{all pauses}}$, and the absolute rate is $100N_k/$final word count. The lower panel uses approximately 347,910 final-text words. These pooled event distributions complement the task-level proportions used in the mixed models.

3 EXPERIMENTAL PROCEDURE AND FEEDBACK CONDITIONS

Table S3. Protocol and intervention parameters.

Domain	Study feature	Operational specification
Keystroke platform	Timestamped action events, cursor movement, and inter-action intervals.	Publicly available WriteTrace keystroke-logging platform, v1.2.3, build 2026-02-26 (`https://writingtrace.com/`); Google Chrome 145 on Windows 11. Study-specific trigger and feedback integration operated around the public platform.
AI provider and model	AI product feedback and AI process feedback were generated by GPT-4o.	GPT-4o (`gpt-4o-2024-11-20`), temperature = 0.2, top_p = 1.0, fully automated generation. The configured maximum output length was 180 tokens.
System architecture	Process feedback used behavioural indicators as diagnostic input.	Rule-based trigger engine with four trigger classes, a 5-minute cooldown, and a maximum of three process-feedback events per task.
Trigger rules	Observable pause, deletion, boundary-activity, and revision indicators triggered process feedback.	Trigger 1: within 90 s, within-clause pause proportion ≥ 0.45 and at least four pauses ≥ 1 s; Trigger 2: within 60 s, at least three delete/replace operations or ≥ 15 deleted characters; Trigger 3: after ≥ 120 words, no sentence/paragraph boundary pause in the preceding 90 s; Trigger 4: within 120 s, at least four modification events without clause-level-or-above revision. The first 5 min were exempt and only the highest-priority rule was executed when multiple rules were satisfied. Event eligibility is specified in Supplementary Table S3a.
Feedback content	Process feedback was diagnostic; product feedback addressed the visible text.	The functional requirements, input fields, output constraints, and distinctions between the two prompt types are described in the section "Feedback Prompt Specifications".

Table S3. Protocol and intervention parameters (continued).

Domain	Study feature	Operational specification
Condition exposure	Product feedback followed the initial draft; process feedback appeared during composing; control received rubric information.	Total feedback events = 773 (191 product, 582 process). Every observed product-feedback task received one message; every observed process-feedback task received three, with 5-minute cooldowns. Individual messages averaged 233.77 words (SD = 22.73, range 175–290) for product feedback and 79.18 words (SD = 6.85, range 64–96) for process feedback. Mean total words per task were 231.08 versus 237.72 at Intervention 1 and 236.55 versus 237.35 at Intervention 2, respectively.
Post-feedback activity	Event records captured latency to the next substantive writing action and whether feedback was accepted fully or partly.	Mean action latency was 91.12 s (SD = 45.85) after product feedback and 62.33 s (SD = 26.35) after process feedback; acceptance or partial acceptance was recorded for 50.8% and 60.3% of events, respectively. An independent blind evaluation covered 382 messages and rated accuracy, actionability, relevance, and potentially misleading content; agreement was weighted κ approximately .79, κ approximately .76, and ICC(2,k) approximately .86, respectively.

Table S3. Protocol and intervention parameters (continued).

Domain	Study feature	Operational specification
Feedback quality evaluation	Independent message-level quality assessment.	All 191 product messages plus a stratified random sample of 191 process messages were rated by two EFL teachers with at least three years of university English-writing teaching experience. Condition, participant, and session labels were removed. Accuracy used accurate/partly accurate/inaccurate; actionability and relevance used 1–5 Likert scales; potentially misleading content used yes/no.
Participants	297 learners were randomized equally to control, product feedback, and process feedback (99 per condition); 279 completed all sessions.	Full-time, non-English-major undergraduates recruited from university English courses at Zhengzhou Sias University from March 2 to March 13, 2026; Chinese as primary language; age 19.98 years (SD = 1.13, range = 18–23); 189 women (63.6%) and 108 men (36.4%); English study 10.4 years (SD = 1.8); prior English writing experience associated with preparation for the National College Entrance Examination (Gaokao); no compensation and no grade effects.
Writing tasks	Four argumentative prompts were assigned through a Latin-square structure.	Exact prompts: (A) community service in the first year; (B) more online rather than face-to-face coursework; (C) larger share of grades for group projects; (D) smartphone restriction in face-to-face classes. Each task allowed 30 min initial writing, 15 min revision, and a 250–350 word target.
Transfer interval	The final task was completed without AI assistance.	Delay between Intervention 2 and transfer: 14 days.

Table S3. Protocol and intervention parameters (continued).

Domain	Study feature	Operational specification
Pause processing	Pause events were extracted from inter-action intervals.	Main threshold: $\geq 1{,}000$ ms. Sensitivity thresholds: 1, 2, 3, and 5 s. Pause locations were coded as within-word, between-word, within-clause, sentence-boundary, or paragraph-boundary under the rules in the process-event coding specification.
Revision coding	Operation, level, function, outcome, delayed status, distance, and feedback relation were coded.	Local revision = character-, word-, or phrase-level change without proposition change; higher-level revision = clause-, sentence-, paragraph-, or discourse-level change. Successful, partly successful, ineffective, and introduced-error rules are defined in the manuscript.
Monitoring task	Twenty-four items required problem identification, location, classification, and 1–5 confidence ratings.	The final instrument comprised six four-item categories, including two no-error items, and was scored 0–24. Pilot KR-20 = .80; study KR-20 = .81, .83, .85, and .82 across baseline, Intervention 1, Intervention 2, and transfer; baseline score correlated with English proficiency at $r = .34$.
English proficiency	Institutionally standardized proficiency measure.	University English Proficiency Test, scored from 0 to 100; overall mean = 67.62 (SD = 9.72), with condition means of 66.56 (SD = 10.10), 68.60 (SD = 10.25), and 67.69 (SD = 8.74) for control, product feedback, and process feedback, respectively.
Writing ratings	Four-dimensional analytic rubric scored from 0 to 100; two blinded university English teachers with three years of teaching experience scored all essays independently.	Raters completed approximately 4–5 h of training and calibrated on 30 essays before formal scoring. The mean of their two total scores formed the outcome; ICC(2,k) = 0.915.
Cognitive load	A post-task 1–7 rating was used.	Single post-task item asking how much mental effort was invested in the writing task, rated from 1 (very low) to 7 (very high).

4 TRIGGER RULES AND EVENT ELIGIBILITY

The four process-feedback rules operated on normalized writing events. Supplementary Table S3a lists the event types used in each trigger calculation and the interface activities assigned to other event classes. The real-time trigger engine and offline process coding drew on the same event stream but were not identical classification procedures. Online triggering used simplified event-level rules based on the current text structure, cursor position, punctuation, and paragraph boundaries. Offline analysis applied the full pause-location and revision-coding protocol to construct statistical variables. When several online rules were satisfied together, the engine executed only the highest-priority rule, followed by a 5-min cooldown; each task admitted at most three process-feedback events.

Table S3a. Treatment of recorded events in the four process-feedback trigger streams.

Recorded event	Trigger treatment
Ordinary character-by-character input	Entered the relevant trigger stream.
Backspace or Delete	Entered the relevant deletion or modification trigger stream.
Ordinary replacement	Entered the relevant replacement or modification trigger stream.
Cursor movement only	Excluded from trigger counts.
Text selection only	Excluded from trigger counts.
Undo or Redo command itself	Excluded from trigger counts.
Net textual change produced by Undo	Coded as an offline revision when a net textual change remained; the original deletion trigger was counted once.
Paste or bulk insertion	Excluded from the production-burst trigger.
Confirmed technical interruption	Excluded from the pause trigger.
Inactivity while the feedback window was displayed	Excluded from the natural-pause trigger.

Table S3b. Representative de-identified example of a process-feedback event.

Element	Representative content
Trigger category	Trigger 1: within-clause pause.
Trigger condition	Within a 90-s window, within-clause pause proportion ≥ 0.45 and at least four pauses $\geq 1{,}000$ ms.
Writing context	The writer is composing an argumentative essay on whether universities should restrict smartphone use during face-to-face classes. The active sentence develops a claim about convenience, frequent classroom use, and learning efficiency.
Observable indicators	Window = 90 s; total detected pauses = 8; pauses $\geq 1{,}000$ ms = 6; within-clause pauses = 5; within-clause proportion = 0.63; current word count = 146; recent deletion events = 1; replacement events = 0; higher-level revision events = 0.
Structured payload	{ "trigger_type": "within_clause_pause", "window_seconds": 90, "pause_count": 8, "pause_ge_1000ms": 6, "within_clause_pause_count": 5, "within_clause_pause_proportion": 0.63, "word_count": 146, "deletion_events": 1, "replacement_events": 0, "higher_level_revision_events": 0 }
Instruction context	Generate concise diagnostic process feedback from observable indicators, avoid cognitive-state inference and text rewriting, and end with a question encouraging learner self-inspection.
Representative output	"You have paused several times while developing a clause in your current sentence. Check whether the relationship between the ideas in this part is clear and whether the sentence structure matches your intended meaning. Which part of the sentence would you like to examine first?"
Feedback action	Diagnostic self-inspection prompt; no replacement sentence or rewritten text.
Retry status	No retry. A retry occurred only after an API timeout, HTTP 429/5xx response, empty output, or format-validation failure. The system waited 2 s and retried once; a second failure was logged as `generation_failed` and was excluded from valid feedback events.

5 WRITING TASK AND STANDARDIZED INSTRUCTIONS

The task instructions specified an English argumentative essay composed for 30 min and revised for a further 15 min. The target length was 250–350 words. Participants stated a position, developed a coherent argument, worked independently in the study interface, and submitted the revised text at the end of the session. External websites, search engines, translation software, electronic dictionaries, and generative-AI tools other than the assigned feedback condition were prohibited.

The four topic prompts were:

1. University students should be required to complete community service in their first year. Discuss this position and present your own argument.
2. A substantial proportion of university coursework should be delivered online rather than fully face-to-face. Discuss this position and present your own argument.
3. Group projects should count for a larger share of university grades. Discuss this position and present your own argument.
4. Universities should restrict smartphone use during face-to-face classes. Discuss this position and present your own argument.

Prompt assignment followed a Latin-square structure across condition and session. Baseline, Intervention 1, and Intervention 2 were separated by 7-day intervals, and the no-AI transfer task followed Intervention 2 after 14 days. The Latin-square allocation balanced prompt exposure across conditions and sessions. Prompt difficulty and equivalence were not formally tested, and prompt fixed effects were included in the statistical models.

6 METALINGUISTIC MONITORING MEASURE

The same 24-item form was administered after the baseline, Intervention 1, Intervention 2, and transfer sessions. Participants received neither the answer key nor item-level correctness feedback after any administration. Repeated exposure could produce item familiarity, so longitudinal inference focused on between-condition differences and condition-by-session change rather than unadjusted within-group score increases. All three conditions followed the same administration schedule.

The monitoring instrument began as a 36-item pool, with six items in each of six categories: grammar/form, lexical choice, sentence structure, cohesion/reference, organization, and argument/discourse. Items were based on recurrent language and discourse problems observed in university EFL argumentative writing and on the operational categories used in the writing and revision coding scheme.

Three experts reviewed the initial pool: two university English/EFL writing teachers and one applied-linguistics/L2-writing researcher, with 8, 11, and 13 years of teaching or research experience. Each expert independently rated item relevance, clarity, category fit, and ambiguity on four-point scales. The reported item-level content-validity indices ranged from .83 to 1.00, and the scale-level average content-validity index (S-CVI/Ave) was .94. Five items were removed because their categories overlapped, and four were reworded, leaving 31 candidates for piloting.

The pilot involved 46 university EFL learners. Across the 31 candidate items, item difficulty ranged from .32 to .78, corrected item–total correlations ranged from .29 to .61, and KR-20 was .80. The final instrument contained 24 items, balanced as four items per category and including two items with no substantive error. For every item, participants identified and located a language or discourse problem, classified its type, and

rated confidence on a 1–5 scale. These four responses were recorded as independent judgements based on the language presented in each item.

Each item received one point when the required identification, location, and classification matched the predetermined answer key. Incorrect and missing responses received zero. Confidence ratings were collected as auxiliary item information and were recorded separately from the primary accuracy score:

$$M_i = \sum_{j=1}^{24} I(\text{item}_{ij} \text{ correct}), \qquad 0 \le M_i \le 24. \tag{S1}$$

No partial-credit weighting was applied. Kuder–Richardson reliability was .81 at baseline, .83 at Intervention 1, .85 at Intervention 2, and .82 at transfer. Baseline monitoring correlated with English proficiency at $r = .34$, $p < .001$. The proficiency association provides convergent evidence, whereas the monitoring task's required identification, localization, and classification of language and discourse problems targets an evaluative skill distinct from a general proficiency score. The instrument was developed for this study and has content-validity, pilot-item, and internal-consistency evidence; independent construct validation against external metalinguistic- knowledge or learning measures was beyond the present measurement package. The complete item set and its predetermined scoring key are presented in Supplementary Table S4. The instrument comprised four items in each of six categories: grammar/form, lexical choice, sentence structure, cohesion/reference, organization, and argument/discourse.

Table S4: Metalinguistic monitoring items, categories, and predetermined answer key.

No.	Item presented to participant	Category	Target location	Correct classification
1	*Many university students believes that online courses are more convenient.*	Grammar/form	**believes**	Subject–verb agreement
2	*The policy was introduced last year, and it have already affected many students.*	Grammar/form	**have**	Verb agreement/form
3	*Students should be encouraged to participated in community service.*	Grammar/form	**participated**	Verb form
4	*Although smartphones are useful, but they can also distract students in class.*	Grammar/form	**Although ... but**	Conjunction/grammatical linkage
5	*The university should make more strict rules about phone use.*	Lexical choice	**make ... rules**	Lexical/collocational choice
6	*Online learning can bring students many conveniences in their study.*	Lexical choice	**conveniences**	Lexical appropriateness
7	*Group work can improve students' ability of communication.*	Lexical choice	**ability of communication**	Collocation/lexical formulation
8	*Students can learn many knowledges from community activities.*	Lexical choice	**knowledges**	Lexical/countability
9	*Students use smartphones during lectures. Which makes it difficult for them to concentrate.*	Sentence structure	**Which makes ...**	Sentence fragment
10	*Because online courses provide greater flexibility.*	Sentence structure	Entire sentence	Sentence fragment
11	*Students who participate in volunteer activities, they may develop stronger social responsibility.*	Sentence structure	**students ... they**	Sentence structure/redundant subject
12	*Group projects are useful students can learn how to cooperate with others.*	Sentence structure	Between **useful / students**	Run-on sentence
13	*University students often use smartphones during class. This problem should be restricted.*	Cohesion/reference	**This problem**	Reference problem
14	*Online courses provide flexibility. However, students can study at any location.*	Cohesion/reference	**However**	Cohesive relation/connective

Continued on next page

Table S4. Metalinguistic monitoring items, categories, and predetermined answer key (continued).

No.	Item presented to participant	Category	Target location	Correct classification
15	*Group work helps students exchange ideas. For example, it also develops their ability to negotiate disagreements.*	Cohesion/reference	**For example**	Incorrect discourse connector
16	*Community service allows students to meet people from different backgrounds. They can improve it through these experiences.*	Cohesion/reference	**it**	Ambiguous/incorrect reference
17	*Smartphones often interrupt classroom attention. They also make information easier to access. Students therefore need clear rules about appropriate classroom use.*	Organization	Ordering of ideas	Organization/logical progression
18	*Online courses save commuting time. Universities should improve campus sports facilities. Online learning also gives students greater scheduling flexibility.*	Organization	Second sentence	Irrelevant sentence/paragraph unity
19	*Group work has several educational benefits. Students can exchange perspectives. Therefore, some students dislike unequal workloads. They can also practise negotiation.*	Organization	**Therefore . . . unequal workloads**	Inappropriate organization/logical transition
20	*Community service can develop social responsibility. It exposes students to community needs. It may also improve interpersonal skills. These reasons show why it can contribute to student development.*	Organization	No target problem	**No substantive discourse problem**
21	*Universities should restrict smartphone use because smartphones are bad.*	Argument/discourse	**smartphones are bad**	Unsupported/underdeveloped claim
22	*Online courses are clearly better than face-to-face courses because students can attend them from home.*	Argument/discourse	**clearly better . . . because . . .**	Insufficient support/overgeneralization
23	*Group projects improve cooperation. For this reason, they should account for 70% of every university course grade.*	Argument/discourse	Inference from first to second sentence	Claim–evidence mismatch/unsupported inference
24	*Community service may help students understand social problems. For example, students working with local organizations can encounter needs that are rarely visible in classroom settings. This experience can provide a basis for reflection on civic responsibility.*	Argument/discourse	No target problem	**No substantive discourse problem**

7 FEEDBACK PROMPT SPECIFICATIONS

7.1 Process-feedback specification

The process-feedback instruction defined the assistant as a diagnostic aid for university EFL writers composing an argumentative essay. Its input consisted of the current process trigger and the supplied observable indicators. The assistant was instructed to identify the most salient process issue represented by that trigger, direct the learner to the relevant part of the ongoing text, give one actionable self-inspection or self-revision direction, and end with a question that required the learner to make the diagnosis or decision. The instruction prohibited inferences about unobserved cognitive states, intentions, emotions, proficiency, or motivation, and it explicitly stated that pausing, deletion, rereading, and revision patterns were not direct evidence of a particular mental operation. It also prohibited replacement sentences, paragraphs, arguments, transitions, rewriting, overall essay scoring, and general evaluation. The prompt requested one English feedback message of approximately 35–60 words, with no explanation of the rule or reasoning. Observed process messages ranged from 64 to 96 words (mean = 79.18). Message length and exposure are summarized in Supplementary Table S3.

7.2 Verbatim process-feedback prompt

```
You are an AI feedback assistant supporting university EFL learners during
↪  argumentative writing. Your role is to provide concise, diagnostic
↪  process feedback that helps learners inspect and regulate their own
↪  writing while composing.

Use only the observable writing-process information provided below. Do not
↪  infer unobserved cognitive states, intentions, emotions, proficiency, or
↪  motivation from the process indicators.

Your task is to identify the most salient writing-process problem indicated
↪  by the current trigger and provide brief guidance that directs the
↪  learner's attention to the relevant aspect of their ongoing writing.

Follow these requirements:

1. Base the feedback only on the observable process indicators supplied.
2. Focus on the specific process problem represented by the trigger.
3. Briefly indicate what aspect of the learner's current writing should be
↪  inspected.
4. Provide one clear and actionable direction for self-inspection or
↪  self-revision.
5. End with one question that encourages the learner to diagnose or
↪  reconsider the relevant issue independently.
6. Do not treat pausing, deletion, rereading, or revision patterns as direct
↪  evidence of a specific cognitive process.
7. Do not provide a replacement sentence, paragraph, argument, transition,
↪  or ready-to-use wording.
8. Do not rewrite any part of the learner's text.
9. Do not provide an overall essay score or a general evaluation of the
↪  entire composition.
10. Keep the feedback concise, approximately 35-60 words.
```

```
11. Use clear English appropriate for a university EFL learner.
12. Return only the feedback message. Do not describe your reasoning or the
↪  triggering rule.

Current process trigger:
{TRIGGER_TYPE}

Observed process indicators:
{PROCESS_INDICATORS}

Generate one concise diagnostic feedback message in English.
```

7.3 Product-feedback specification

The product-feedback instruction defined the assistant as a diagnostic aid used after the initial argumentative draft. Its primary input was the student draft. The requested review covered language accuracy, organization, coherence, and revision priorities; the assistant was to identify the most important visible problems, explain what the learner should inspect, and give actionable guidance for independent revision. The instruction excluded keystroke behaviour, pauses, deletions, revision timing, and other process information. It prohibited complete replacement sentences or paragraphs, ready-made arguments, substantial reproduction of corrected text, and unnecessary diagnostic information. The output was concise English diagnostic feedback only, without a reasoning trace.

7.4 Verbatim product-feedback prompt

```
You are an AI feedback assistant supporting university EFL learners in
↪  revising an argumentative essay after completion of an initial draft.
↪  Your role is to provide concise diagnostic feedback on the written
↪  product so that the learner can revise the draft independently.

Review only the student draft provided below. Do not use, request, or infer
↪  information about the learner's keystroke behavior, pausing, deletion
↪  patterns, revision timing, or other writing-process indicators.

Evaluate the draft with attention to the following areas:

- language accuracy;
- organization;
- coherence;
- revision priorities.

Follow these requirements:

1. Identify the most important problems that are visible in the current
↪  draft.
2. Prioritize issues that are likely to have the greatest effect on the
↪  quality of the revised text.
3. Briefly explain what the learner should inspect or reconsider.
4. Provide clear and actionable diagnostic guidance for revision.
5. Focus on helping the learner identify and address problems independently.
```

```
6. Do not rewrite complete sentences or paragraphs.
7. Do not provide replacement sentences, replacement paragraphs, ready-made
↪  arguments, or ready-to-submit text.
8. Do not substantially reproduce the learner's text in corrected form.
9. Do not introduce information that is not necessary for diagnosing the
↪  draft.
10. Use clear English appropriate for a university EFL learner.
11. Keep the feedback concise.
12. Return only the diagnostic feedback. Do not describe your reasoning
↪  process.

Student draft:
{STUDENT_DRAFT}

Generate concise diagnostic feedback in English.
```

The feedback service was integrated with the publicly available WriteTrace platform, v1.2.3 (build 2026-02-26), in Google Chrome 145 on Windows 11. API timeouts, HTTP 429/5xx responses, empty responses, and format-validation failures triggered one automatic retry after 2 s. A second failure produced no substitute human message, was recorded as `generation_failed`, and did not count as a successful feedback event; a later qualifying trigger could generate a new request. Outputs that directly rewrote a student's sentence, addressed an unrelated issue, were empty, or clearly exceeded the configured length limit were discarded and retried once.

7.5 Independent feedback-quality evaluation

The independent quality evaluation covered 382 messages: all 191 product-feedback messages and a stratified random sample of 191 of the 582 process-feedback messages, sampled across Intervention 1 and Intervention 2. Two EFL teachers with at least three years of university English-writing teaching experience rated the messages independently after condition, participant, and session labels had been removed. Accuracy was coded as accurate, partly accurate, or inaccurate. Actionability and relevance were rated on five-point Likert scales, and potentially misleading feedback was coded yes/no. Agreement was weighted Cohen's $\kappa = .79$ for accuracy, Cohen's $\kappa = .76$ for the potentially misleading-feedback judgment, ICC(2,k) = .86 for actionability, and ICC(2,k) = .83 for relevance. Accurate ratings accounted for 85.3% of product feedback and 88.5% of process feedback; potentially misleading content occurred in 4.2% and 3.1% of messages, respectively. Mean relevance was 4.35 for product feedback and 4.41 for process feedback, and mean actionability was 4.29 and 4.47. Supplementary Table S3c reports the complete category counts and five-point rating distributions.

7.6 Feedback uptake coding

Feedback uptake was coded for each of the 773 feedback events as full uptake, partial uptake, or no observable uptake. The coding unit was the observable response in the writing log: full uptake required a substantive revision that addressed the principal feedback target, whereas partial uptake indicated identifiable inspection or revision addressing part of that target. No observable uptake indicated that the log contained no target-related inspection or revision during the coding window. The coding used the feedback text, presentation time, text snapshots, cursor position, and insertion, deletion, replacement, and revision events. For process feedback, the window began when the feedback display closed and ended at the next process message, 5 min, or the end of the composing phase. For product feedback, the window comprised

the first 5 min of the revision phase. Events beyond these windows were excluded from the primary uptake rates.

All events were coded by one trained research coder using the coding manual. A second coder independently reviewed 232 events (30.0%), stratified by condition, intervention session, and process-feedback trigger class. After 60 separate calibration cases, the three-category agreement rate was 87.9% (204/232), with weighted Cohen's $\kappa = .82$, 95% CI [.76, .88]. When full and partial uptake were combined, agreement was 93.1% (216/232), with Cohen's $\kappa = .86$, 95% CI [.79, .93]. Twenty-eight disagreements were adjudicated by a senior L2-writing researcher. Final rates were based on the adjudicated coding. Full, partial, and no-observable-uptake counts were 43, 54, and 94 for product feedback, and 164, 187, and 231 for process feedback, respectively; thus observable uptake occurred in 50.8% and 60.3% of the two event sets (Supplementary Table S3d).

Table S3c. Independent blind evaluation of product and process feedback.

Quality indicator	Product feedback ($n = 191$)	Process feedback ($n = 191$)	Total ($N = 382$)
Accurate	163 (85.3%)	169 (88.5%)	332 (86.9%)
Partly accurate	24 (12.6%)	19 (9.9%)	43 (11.3%)
Inaccurate	4 (2.1%)	3 (1.6%)	7 (1.8%)
Potentially misleading: yes	8 (4.2%)	6 (3.1%)	14 (3.7%)
Potentially misleading: no	183 (95.8%)	185 (96.9%)	368 (96.3%)
Relevance, mean (SD)	4.35 (0.79)	4.41 (0.70)	4.38 (0.75)
Actionability, mean (SD)	4.29 (0.82)	4.47 (0.66)	4.38 (0.75)

Rating	Relevance: product	Relevance: process	Actionability: product	Actionability: process
1	1	0	1	0
2	4	3	5	2
3	20	14	24	12
4	68	75	69	71
5	98	99	92	106

Two EFL teachers independently rated messages after condition, participant, and session labels had been removed; a third senior EFL teacher adjudicated disagreements. Agreement was weighted Cohen's $\kappa = .79$ for accuracy, Cohen's $\kappa = .76$ for potentially misleading content, ICC(2,k) = .86 for actionability, and ICC(2,k) = .83 for relevance.

Table S3d. Observable feedback uptake by intervention package.

Feedback condition	Full	Partial	No observable	Full + partial
Product feedback ($n = 191$)	43 (22.5%)	54 (28.3%)	94 (49.2%)	97 (50.8%)
Process feedback ($n = 582$)	164 (28.2%)	187 (32.1%)	231 (39.7%)	351 (60.3%)

8 PROCESS MEASURES AND CODING RULES

8.1 Action normalization and exclusions

Single-character insertions were treated as production events, and consecutive production entered the production-burst calculation. Backspace and Delete actions were treated as deletion events; consecutive deletions affecting the same text region and separated by no more than 2 s were merged into one revision episode. When selected text was deleted and immediately replaced, the sequence was coded as one replacement revision instead of separate deletion and insertion revisions.

Paste activity was identified from an explicit `insertFromPaste` browser event or from an input event that added at least two characters without corresponding character-by-character keystrokes. Such events were marked `PASTE/BULK_INSERT`; pasted characters were excluded from character-by-character production and production-burst length. A paste that replaced existing text formed one revision episode, with its level determined by the final scope of the textual change. Where an external source could be established, the task was marked as a protocol violation because external resources were prohibited. A paste whose source could not be determined was classified as `bulk_insert_source_unknown`. Cut-and-paste movement within the same text was coded as relocation rather than new production. Relocation of a complete clause, sentence, or paragraph could be classified as a higher-level revision according to the resulting textual change.

`historyUndo`, Ctrl+Z, and corresponding redo actions were treated as state reversals and were excluded from independent revision counts. An undo–redo cycle that fully restored the preceding text state contributed zero net revision. When a post-undo state remained, its final net textual change was coded once. Cursor-only movement and selection without textual modification were classified as navigation and were excluded from production, revision, deletion, and modification trigger counts. Cursor and selection positions were used to locate edits, calculate revision distance, and assign pause location.

Technical interruption was coded from explicit blur/focus, disconnect, reload, crash, or observer records. Confirmed interruption time was removed from pause-duration and burst-duration calculations. For a brief interruption with a continuous log and recoverable text state, the interruption interval alone was removed. An unrecoverable text state, critical log segment, or required task restart produced an incomplete process record, with no keystroke imputation. Feedback display was represented as a separate state, and its inactivity interval was excluded from natural composing pauses. Autosave, interface refresh, and other non-text DOM or system events were excluded from production, revision, and pause counts.

8.2 Pause detection and location

A pause event was identified from the elapsed time between consecutive recorded actions. The primary threshold was 1,000 ms; pause-count models were repeated at 1-, 2-, 3-, and 5-s thresholds. The primary dataset contained 106,470 pause events, every analysed task contained at least one pause event, and the audit found no invalid normalized pause times.

Pause location was assigned from the cursor position in the evolving text. The five categories were within-word, between-word, within-clause, sentence-boundary, and paragraph-boundary. A within-word pause occurred inside an unfinished lexical token, with subsequent typing continuing to form the same word. A between-word pause occurred between two complete words while the same local syntactic constituent continued. A within-clause pause occurred inside an unfinished clause but outside a within-word position, including positions inside a phrase or constituent and clause-internal positions following a comma. A sentence-boundary pause required a completed sentence-final boundary, with the cursor at the end of that

sentence and subsequent production beginning a new sentence. A paragraph-boundary pause required the cursor to be at the end of a completed paragraph and subsequent substantive production to begin a new paragraph. If more than one structural description applied, the larger textual boundary took precedence:

$$\text{paragraph-boundary} > \text{sentence-boundary} > \text{within-clause} > \text{between-word} > \text{within-word}. \quad \text{(S2)}$$

The two principal location outcomes were

$$P_{WC} = \frac{N_{WC}}{N_{\text{pause}}}, \quad \text{(S3)}$$

$$P_B = \frac{N_{SB} + N_{PB}}{N_{\text{pause}}}. \quad \text{(S4)}$$

These variables describe the textual distribution of inactivity during composing.

8.3 Production bursts

A production burst comprised words entered during continuous text production between qualifying pauses. Cursor movement without text insertion contributed no words. For task t, mean burst length was calculated as

$$\overline{B}_t = \frac{\sum_{b=1}^{B_t} W_{bt}}{B_t}, \quad \text{(S5)}$$

where B_t is the number of bursts and W_{bt} is the number of words produced in burst b. The 1,000-ms pause threshold defined bursts in the primary analysis.

8.4 Revision identification, level, and outcome

A revision event occurred when insertion, deletion, replacement, or movement modified text that had already been produced. Character-, word-, and phrase-level changes were classified as local when the clause proposition was preserved. Higher-level revisions affected a clause, sentence, paragraph, or discourse relation, including propositional content, paragraph development, argument structure, and claim–evidence relations. The task-level proportion was

$$P_{HLR} = \frac{N_{clause} + N_{sentence} + N_{paragraph} + N_{discourse}}{N_{revision}}. \quad \text{(S6)}$$

Revision outcomes were coded as successful, partly successful, ineffective, or introduced-error. A successful revision resolved the target without creating another problem; a partly successful revision improved the target but left a residual problem; an ineffective revision did not improve the target; and an introduced-error revision created a new linguistic or discourse problem. Successful and partly successful events formed the effective-revision outcome:

$$P_{ER} = \frac{N_{successful} + N_{partly\ successful}}{N_{revision}}. \quad \text{(S7)}$$

A delayed revision returned to earlier text after intervening production, whereas an immediate revision modified the current production location before intervening text was entered. Revision distance recorded the cursor displacement in characters from the pre-navigation production position to the start of the revised

span, with backward or forward direction identified. Delayed status and distance were complementary process measures. Of 23,374 revision events, 5,844 (25.0%) were independently double-coded by two human coders after a 300-event calibration stage. Sampling was stratified by condition and study stage; unresolved disagreements were adjudicated by a third senior EFL-writing researcher. Cohen's κ was .783 for revision level and .764 for revision effectiveness.

9 OUTCOME HIERARCHY AND ANALYSIS PLAN

The analysis hierarchy designated pause-location trajectories, higher-level revision, effective revision, and transfer ANCOVA as primary outcomes. Mean production-burst length, transfer monitoring, mediation, and state-transition measures were secondary outcomes. Alternative pause thresholds were sensitivity analyses, while feedback-window and state-transition analyses were exploratory analyses focused on activity surrounding feedback and dynamic organization of writing states, respectively.

Table S5. Outcome hierarchy and analysis mapping used in the study.

Analysis tier	Outcomes	Primary comparison	Analysis scope
Primary process outcomes	Within-clause pause proportion and boundary pause proportion.	Condition by session contrasts relative to control and baseline.	Primary process outcomes in the repeated-measures trajectory analyses.
Secondary process outcome	Mean production-burst length.	Condition by session contrasts and transfer comparison.	Secondary process outcome describing continuity of text production.
Primary revision outcomes	Higher-level revision proportion and effective revision proportion.	Condition by session contrasts, with transfer included as a no-AI outcome.	Revision outcomes in the repeated-measures and transfer analyses.
Complementary revision outcomes	Delayed revision status and absolute revision distance; introduced error as a descriptive supplementary outcome.	Direct process-versus-product contrasts at Intervention 2 and transfer.	Location, temporal delay, and outcome of revision beyond revision frequency.
Sensitivity outcome	Total pause count under alternative thresholds.	Negative-binomial GEE contrasts.	Sensitivity analysis separating pause-event counts from pause-location proportions.
Exploratory window analysis	Pause and higher-level-revision rates around process-feedback triggers.	Pre-feedback versus post-feedback windows.	Window-level characterization of activity surrounding feedback presentation.
Exploratory state analysis	Transition probabilities among processing states.	Aggregate process-feedback versus control differences.	Dynamic summary of transitions across 20 fixed time bins.
Transfer and indirect associations	Transfer quality, metalinguistic monitoring, higher-level revision, and bootstrap indirect estimates.	Baseline-adjusted group contrasts and observed mediator associations.	Transfer contrasts and statistical pathway estimates are reported separately.

10 DEFINITIONS AND CODING OF ANALYSIS VARIABLES

Table S6. Definitions and coding of variables used in the analyses.

Variable domain	Definition and coding
Participant characteristics	Participants were classified by experimental condition: control, product feedback, or process feedback. Baseline covariates were age, recorded gender, English proficiency, typing speed, and prior AI use.
Task and stage	Each writing observation was identified by participant, study stage, and prompt. Stages were baseline, Intervention 1, Intervention 2, and transfer; task-level outcomes included word count, writing quality, cognitive load, and metalinguistic monitoring.
Keystroke actions	Writing-process events included text insertion, deletion, replacement, cursor movement, selection, event time, character class, affected span, inter-action interval, and cursor position. Event normalization distinguished paste or bulk insertion, relocation, undo or redo, technical interruption, and non-text interface activity.
Pause location	Pauses were classified as within-word, between-word, within-clause, sentence-boundary, or paragraph-boundary. The principal location outcomes were within-clause pause proportion and boundary pause proportion.
Production bursts	Mean production-burst length was the mean number of words produced between qualifying pauses. Pasted characters and cursor-only movement were excluded from burst length.
Revision level and outcome	Revision events were classified by linguistic level, operation, outcome, delayed status, revision distance, and relation to feedback. Local revision covered character, word, and phrase changes without proposition change; higher-level revision covered clause, sentence, paragraph, and discourse changes.
Revision effectiveness	Successful and partly successful revisions formed the effective-revision outcome. Ineffective and introduced-error revisions remained separate outcome categories.
Feedback exposure	Process feedback was linked to trigger class, presentation time, message length, action latency, and uptake. Product feedback was delivered after the initial draft, whereas process feedback was delivered during composing.
State transitions	Transitions among planning, production, monitoring, local revision, and global revision were summarized across 20 fixed time bins.
Missing covariates	Missing typing-speed and prior-AI-use values were replaced by the corresponding sample medians, and missingness indicators were included in relevant models.
Measurement documentation	The Supplementary Material specifies the task instructions, measurement procedures, coding rules, scoring criteria, and analysis mappings used for the reported outcomes.

11 AMBIGUOUS CASES IN PROCESS CODING

The decision matrix in Supplementary Table S6a applies the event-time text state, actual cursor position, and final net textual change to cases in which surface punctuation or interface actions could otherwise produce more than one code.

Table S6a: Representative decision rules for ambiguous pause, navigation, revision, paste, and interruption events.

Observed event pattern	Coding decision
Punctuation added only after a pause	The pause was coded from the text state when it occurred; a subsequently inserted full stop did not retrospectively convert it to a sentence-boundary pause.
Full stop entered, deleted within 1–2 s, and the same sentence continued	When the full stop was a transient attempt and no new production episode began, the event was coded from the final continuous structure and classified with a `transient_boundary` flag.
Full stops in *e.g.*, *i.e.*, or decimal numbers	These punctuation marks were not coded as sentence boundaries.
Pause after a comma	The sentence remained evolving; location was assigned from the contemporaneous syntactic structure and coded within-clause (clause-internal) when the clause was unfinished.
Pause after typing *because*	The incomplete subordinate structure remained an evolving clause and was coded within-clause.
Cursor moved to an earlier sentence and text was changed	Revision location was assigned from the cursor's actual edit position, not from the endpoint of the most recently produced text.
Cursor moved without text modification	The event was classified as navigation and did not count as a revision.
An entire sentence was selected and replaced	The sequence counted as one sentence-level higher-level revision.
One word was deleted and immediately retyped identically	If no net textual change remained, the sequence counted as one revision episode coded ineffective (mechanical retyping), rather than as separate deletion and insertion events.
A sentence was deleted and then restored by Undo	The deletion and restoration were not counted as two higher-level revisions; when the net state was unchanged, no persistent revision was recorded.
A complete sentence was pasted into an existing paragraph	Pasted text did not contribute to production-burst length. When it changed the text, the event was coded as a sentence-level insertion marked `PASTE/BULK_INSERT`.
A sentence was cut and moved to another paragraph	The move was coded as relocation and ordinarily as a paragraph- or discourse-level revision according to its effect on organization or argument relations.
Cursor moved 30 characters backward to correct spelling	The event was coded as a local revision, and revision distance was recorded as a 30-character backward movement.

Continued on next page

Supplementary Table S6a continued

Observed event pattern	Coding decision
Cursor returned to an earlier paragraph to change an argument relation	The event was coded as a higher-level revision.
A feedback window produced 60 s without typing	Feedback-display inactivity was excluded from natural composing pauses.
A documented network disconnection produced 90 s without typing	When disconnect, blur/focus, or equivalent technical evidence was present, the 90-s interval was removed from pause-duration calculations.
Ninety seconds without typing and without technical evidence	The interval remained behavioural inactivity and was not automatically reclassified as a technical interruption.

12 SAMPLE RETENTION, ATTRITION, AND DATA QUALITY

The 297 randomized participants contributed 297 baseline, 292 Intervention 1, 285 Intervention 2, and 279 transfer records, for 1,153 task-level observations. Longitudinal mixed-effects and generalized estimating equation (GEE) models used all available task records and accounted for repeated observations through participant random intercepts or participant-clustered robust standard errors. Transfer ANCOVA and sequential mediation used the 279 participants who completed the transfer sequence (93 control, 92 product feedback, and 94 process feedback). No task-level outcome was imputed.

Completion rates were 93.9% in control, 92.9% in product feedback, and 94.9% in process feedback. Table S7 compares participants who completed all sessions with participants with incomplete follow-up. Completers had higher baseline English proficiency, writing quality, and metalinguistic monitoring, with standardized mean differences of 2.087, 1.779, and 1.701, respectively. Completion rates were similar across the three conditions, while baseline performance differed between completers and incomplete cases.

Data-quality screening covered participant and task identifier validity, session ordering, process-record completeness, event chronology, nonnegative inter-action intervals, pause-location and revision-code validity, feedback-session eligibility, writing-score range, and baseline-covariate completeness. Three participants had missing typing-speed values and 10 had missing prior-AI-use values. These small numbers of covariate omissions were median imputed to preserve the analytic sample, with separate missingness indicators included in the relevant models. The median was selected as a robust location estimate for the affected covariates and avoided model-derived values for the missing observations. Longitudinal models used all available task records; no task-level outcome was imputed.

13 SUPPLEMENTARY STATISTICAL RESULTS

Tables S8–S11 report absolute revision activity, feedback-window estimates, state-transition uncertainty, and a median-burst sensitivity summary. The analyses use the event-level and participant-level observations described above.

Table S7. Baseline characteristics of participants who completed all sessions and those with incomplete follow-up.

Measure	Completers (n=279)	Incomplete (n=18)	Difference	SMD
Age, years	20.000 (1.138)	19.722 (1.018)	0.278	0.246
Female, n (%)	174 (62.4)	15 (83.3)	-21.0 pp	-0.485
English proficiency	68.719 (8.890)	50.533 (5.060)	18.186	2.087
Typing speed, WPM	37.330 (8.537)	32.006 (9.461)	5.324	0.620
Prior AI use, 0–5	2.793 (1.131)	1.765 (1.147)	1.028	0.908
Baseline writing quality	65.062 (8.610)	50.012 (5.398)	15.049	1.779
Baseline monitoring score, 0–24	12.452 (4.561)	4.833 (2.813)	7.618	1.701
Within-clause pause proportion	0.285 (0.052)	0.286 (0.049)	-0.001	-0.013
Higher-level revision proportion	0.160 (0.106)	0.120 (0.066)	0.040	0.386

Values are mean (SD) unless stated otherwise. Differences are completers minus incomplete cases; pp denotes percentage points. SMD denotes the standardized mean or proportion difference using the pooled standard deviation. Typing-speed values were available for 276 completers and all 18 incomplete cases; prior-AI-use values were available for 270 completers and 17 incomplete cases. The table uses observed baseline values before imputation.

Table S8. Direct process-versus-product revision-count checks.

Session	Condition	n	Total revisions per task	Higher-level revisions per task
Intervention 2	Product feedback	94	22.60	6.07
Intervention 2	Process feedback	96	22.90	6.46
Transfer	Product feedback	92	20.60	4.18
Transfer	Process feedback	94	20.86	5.07

Analysis	Session	Contrast	Estimate (95% CI)	p value
Absolute higher-level event-count IRR	Intervention 2	Process vs. product	1.06	.251
Absolute higher-level event-count IRR	Transfer	Process vs. product	1.20 [1.08, 1.34]	.001
Total revision activity IRR	Transfer	Process vs. product	approximately 1.02	approximately .59
Total-revision-adjusted higher-level ratio	Intervention 2	Process vs. product	1.08 [0.95, 1.23]	.239
Total-revision-adjusted higher-level ratio	Transfer	Process vs. product	1.30 [1.11, 1.53]	.001

The analysis included 23,374 total revision events. The first table reports task-level means for the direct process-versus-product count check; the absolute higher-level differences were +0.39 events per task at Intervention 2 and +0.89 at transfer. The absolute higher-level IRR is an unadjusted process-minus-product count contrast. The total-revision-adjusted ratio includes total revision activity as a covariate and is reported alongside the primary higher-level revision-proportion model. Approx. denotes values supplied to two significant figures.

Table S8a. Delayed revision, absolute revision distance, and introduced-error outcomes.

Session	Condition	Delayed revision	Absolute distance, median [IQR], characters	Introduced error
Intervention 2	Control	24.9%	36 [14–82]	7.8%
Intervention 2	Product	27.8%	41 [16–96]	6.9%
Intervention 2	Process	31.9%	48 [19–114]	5.4%
Transfer	Control	24.2%	35 [13–81]	7.6%
Transfer	Product	25.6%	38 [15–89]	7.3%
Transfer	Process	32.8%	52 [20–123]	5.8%

Outcome	Contrast	Intervention 2	Transfer
Delayed revision	Process vs. product	OR = 1.21 [1.06, 1.39], $p = .005$	OR = 1.42 [1.23, 1.64], $p < .001$
Absolute revision distance	Process vs. product	Ratio = 1.13 [1.03, 1.24], $p = .009$	Ratio = 1.27 [1.14, 1.41], $p < .001$
Introduced error	Process vs. product	OR = 0.77 [0.59, 1.01], $p = .061$	OR = 0.78 [0.59, 1.04], $p = .091$

Delayed revision indicates a return to an earlier location after new intervening text had been produced. Absolute revision distance is the character displacement from the pre-revision production position to the start of the revised span. Introduced error identifies a revision that created a new language or discourse problem. Bracketed values in the lower panel are 95% confidence intervals.

Table S9. Participant-cluster bootstrap intervals for primary and alternative feedback-window summaries.

Session	Window	Events	Participants	Mean rate/min	Bootstrap 95% CI
Intervention 1	Pre 3 min	294	98	0.120	[0.108, 0.132]
Intervention 1	Post 0–1 min	294	98	0.111	[0.099, 0.123]
Intervention 1	Post 1–3 min	294	98	0.122	[0.109, 0.135]
Intervention 1	Post 3–5 min	294	98	0.141	[0.126, 0.156]
Intervention 2	Pre 3 min	288	96	0.122	[0.109, 0.135]
Intervention 2	Post 0–1 min	288	96	0.130	[0.116, 0.144]
Intervention 2	Post 1–3 min	288	96	0.151	[0.136, 0.166]
Intervention 2	Post 3–5 min	288	96	0.182	[0.164, 0.200]
Intervention 1	Pre 4 min	294	98	0.114	[0.095, 0.134]
Intervention 1	Post 0–2 min	294	98	0.107	[0.083, 0.131]
Intervention 1	Post 2–4 min	294	98	0.136	[0.100, 0.172]
Intervention 1	Post 4–6 min	294	98	0.163	[0.131, 0.196]
Intervention 2	Pre 4 min	288	96	0.119	[0.097, 0.141]
Intervention 2	Post 0–2 min	288	96	0.141	[0.113, 0.170]
Intervention 2	Post 2–4 min	288	96	0.163	[0.128, 0.200]
Intervention 2	Post 4–6 min	288	96	0.191	[0.151, 0.231]

The first block uses the primary 3-min pre-feedback reference and 0–1, 1–3, and 3–5-min post-feedback windows to distinguish immediate, short-term, and later within-task activity. The second block is an alternative 4-min-window specification. The exact cut-points were not preregistered; they were defined as a temporal framework and evaluated with alternative windows. Intervals are participant-level bootstrap percentile intervals from 2,000 resamples; rates are higher-level revision events per minute. The 191 product-feedback events were not included in these composing-window summaries. In an additional matched analysis, 566 actual-feedback events were paired with pseudo-trigger events matched on session, trigger class, elapsed time, and pre-trigger activity. The original higher-level-revision estimate was +0.026 [0.004, 0.047] ($p = .020$), the pseudo-trigger natural change was +0.013, and the adjusted feedback effect was +0.016 [0.003, 0.029] ($p = .016$). The adjusted immediate pause effect was -0.058 [-0.098, -0.018] ($p = .005$). Alternative windows at 2–4, 3–5, and 4–6 min produced higher-level-revision sensitivity effects from +0.012 to +0.016. The matched analysis used participant-clustered comparisons with 2,000 bootstrap resamples.

Table S10. Participant-level bootstrap intervals for Intervention 2 state-transition probability differences.

From state	To state	Δ probability	Bootstrap 95% CI	BH-FDR q
Planning	Planning	-0.020	[-0.061, 0.020]	.520
Planning	Production	+0.040	[-0.008, 0.087]	.309
Planning	Monitoring	-0.030	[-0.074, 0.014]	.368
Planning	Local revision	0.000	[-0.036, 0.035]	1.000
Planning	Global revision	+0.010	[-0.025, 0.046]	.807
Production	Planning	0.000	[-0.029, 0.028]	1.000
Production	Production	-0.072	[-0.116, -0.030]	.013
Production	Monitoring	+0.030	[-0.009, 0.069]	.329
Production	Local revision	0.000	[-0.034, 0.035]	1.000
Production	Global revision	+0.041	[-0.008, 0.089]	.309
Monitoring	Planning	+0.030	[-0.016, 0.074]	.368
Monitoring	Production	-0.030	[-0.075, 0.015]	.368
Monitoring	Monitoring	+0.010	[-0.035, 0.056]	.838
Monitoring	Local revision	-0.020	[-0.061, 0.020]	.520
Monitoring	Global revision	+0.023	[-0.028, 0.072]	.540
Local revision	Planning	0.000	[-0.031, 0.031]	1.000
Local revision	Production	-0.067	[-0.121, -0.015]	.110
Local revision	Monitoring	+0.010	[-0.036, 0.056]	.838
Local revision	Local revision	-0.057	[-0.110, -0.006]	.158
Local revision	Global revision	+0.118	[+0.052, +0.181]	.008
Global revision	Planning	0.000	[-0.037, 0.038]	1.000
Global revision	Production	-0.060	[-0.126, 0.005]	.302
Global revision	Monitoring	+0.031	[-0.021, 0.083]	.433
Global revision	Local revision	-0.050	[-0.112, 0.012]	.317
Global revision	Global revision	+0.082	[+0.010, +0.151]	.141

The analysis used 95 control and 96 process-feedback participants and 20 fixed time bins. Values are aggregate transition probabilities; intervals are percentile intervals from 2,000 participant-level bootstrap resamples. The Benjamini–Hochberg procedure was applied across the 25-transition family. Production-to-production and local-revision-to-global-revision remained supported after correction. Global-revision persistence had a positive bootstrap interval but $q = .141$ and is reported descriptively.

Table S11. Descriptive sensitivity summary using task-level median production-burst length.

Session	Condition	n	Mean task-level median (words)	SD
Baseline	Control	99	3.722	1.457
Baseline	Product feedback	99	4.091	1.409
Baseline	Process feedback	99	3.667	1.284
Intervention 1	Control	97	3.933	1.328
Intervention 1	Product feedback	97	4.232	1.463
Intervention 1	Process feedback	98	4.184	1.422
Intervention 2	Control	95	4.205	1.661
Intervention 2	Product feedback	94	4.324	1.773
Intervention 2	Process feedback	96	4.464	1.782
Transfer	Control	93	4.468	1.904
Transfer	Product feedback	92	4.163	1.420
Transfer	Process feedback	94	4.404	1.561

Values are task-level means of the within-task median number of words per production burst. The table provides a descriptive median-based sensitivity summary for the primary mean-burst outcome; the longitudinal inferential model used mean burst length on the logarithmic scale. The median summary showed the same directional process-feedback pattern at Intervention 2 and transfer, with higher values than product feedback in both sessions.

14 MODEL DIAGNOSTICS AND EXPLORATORY ANALYSES

Continuous-outcome mixed models used a participant random intercept and fixed effects for prompt, condition, session, and the condition-by-session interaction. Standardized residual-versus-fitted plots, residual Q–Q plots, and influential observations were inspected; no convergence or singular-fit warning was recorded. Pause counts were overdispersed relative to a Poisson model and were therefore fitted with negative-binomial GEE. The principal GEE models used an exchangeable working correlation and robust sandwich standard errors; refitting the focal contrasts under an independence working correlation did not alter their direction or interpretation.

The secondary transfer-monitoring comparison used an HC3-robust linear model with transfer monitoring as the outcome and baseline monitoring, feedback condition, English proficiency, typing speed, typing-speed missingness, and transfer-prompt assignment as predictors. The direct process-versus-product contrast was 2.442 score points (95% CI [1.784, 3.100], $p < .001$).

No study-wide multiplicity adjustment was applied. Reported p values are nominal, two-sided values. Interpretation therefore follows the outcome hierarchy in Supplementary Table S5: pause-location and revision trajectories and transfer ANCOVA provide the primary comparisons, whereas pause-threshold, feedback-window, mediation, and state-transition analyses are exploratory. Effect estimates, 95% confidence intervals, and estimation precision are reported alongside p values.

Feedback windows used the 3 min before presentation as the local reference and divided post-feedback time into 0–1, 1–3, and 3–5 min intervals. Each of the 582 process-feedback events contributed all four records, yielding 2,328 window-level observations; the 191 post-draft product-feedback events were excluded from this composing-window analysis. Adjacent process-feedback events were more than 5 min apart, so their post-feedback windows did not overlap. For 96 of the 388 adjacent within-task event pairs, the next event occurred within 8 min, allowing its 3-min pre-feedback interval to overlap the preceding event's later post-feedback interval. Records remained indexed to their focal event, and the GEE models clustered observations by participant.

14.1 Pseudo-trigger construction and matching

The trigger engine was replayed offline on control-condition Intervention 1 and Intervention 2 logs using the same first-5-min exemption, four trigger rules, priority rule, 5-min cooldown, and maximum of three eligible events per task. This procedure identified approximately 572 eligible pseudo-trigger candidates. Matching required the same session and trigger class and elapsed task time within ± 90 s. Standardized Euclidean nearest-neighbour matching then used within-clause pause proportion, pause rate per minute, delete/replace count, higher-level revision rate, and cumulative word count during the preceding 3 min. Matching was conducted without replacement with a maximum standardized-distance caliper of 0.75 SD. Of 582 actual process-feedback events, 566 obtained one-to-one matches (97.3%); 16 had no candidate within the caliper. In the matched sample, the original Intervention 2 higher-level-revision estimate was +0.026 (95% CI [0.004, 0.047], $p = .020$), the pseudo-trigger change was +0.013, and the adjusted feedback contrast was +0.016 (95% CI [0.003, 0.029], $p = .016$). The adjusted immediate pause contrast was -0.058 (95% CI [-0.098, -0.018], $p = .005$).

14.2 Time-bin state assignment

The 30-min initial composing phase of Intervention 2 was divided into 20 fixed 90-s bins. Each bin was assigned to the behaviour occupying the largest share of valid behavioural time. Planning comprised

qualifying inactivity at the current text endpoint followed by resumed new-text production without intervening navigation or revision. Production comprised character-by-character new-text generation. Monitoring comprised cursor navigation, selection, or rereading-related inactivity over existing text without textual modification. Local revision comprised character-, word-, or phrase-level modification, and global revision comprised clause-, sentence-, paragraph-, or discourse-level modification; global revision therefore used the same textual-level boundary as higher-level revision elsewhere in the manuscript. Feedback-display time and confirmed technical interruptions were removed from the valid-time denominator. Exact ties were resolved in the order global revision, local revision, monitoring, production, and planning. Transitions were constructed only between adjacent classifiable bins and did not cross missing or unclassifiable bins. Supplementary Table S10 reports participant-level bootstrap intervals and BH-FDR q values for all 25 transition contrasts.
